# DNA methylation mediates the association between breastfeeding and early-life growth trajectories

**DOI:** 10.1186/s13148-021-01209-z

**Published:** 2021-12-22

**Authors:** Laurent Briollais, Denis Rustand, Catherine Allard, Yanyan Wu, Jingxiong Xu, Samyukta Govinda Rajan, Marie-France Hivert, Myriam Doyon, Luigi Bouchard, Patrick O. McGowan, Steven Matthews, Steven Lye

**Affiliations:** 1 Lunenfeld-Tanenbaum Research Institute, Sinai Health System, 60, Murray street – Room 5-237, Toronto, ON M5T 3L9 Canada; 2 Dalla Lana School of Public Health, University of Toronto, Toronto, ON Canada; 3 Biostatistics Team, Bordeaux Population Health Center, ISPED, Centre INSERM U1219, Bordeaux, France; 4 Centre de Recherche du Centre Hospitalier Universitaire de Sherbrooke (CHUS), Sherbrooke, QC Canada; 5 Department of Public Health Sciences, University of Hawai’i at Manoa, Honolulu, HI USA; 6 Division of Chronic Disease Research Across the Life Course, Department of Population Medicine, Harvard Pilgrim Health Care Institute, Harvard Medical School, Boston, MA 02215 USA; 7 Department of Medicine, Université de Sherbrooke, Sherbrooke, QC J1H 5N4 Canada; 8 Diabetes Unit, Massachusetts General Hospital, Boston, MA 02114 USA; 9 Department of Medical Biology, CIUSSS Saguenay-Lac-Saint-Jean, Hôpital Universitaire de Chicoutimi, Saguenay, QC G7H 5H6 Canada; 10 Department of Biochemistry and Functional Genomics, Université de Sherbrooke, Sherbrooke, QC J1K 2R1 Canada; 11 Department of Biological Sciences, University of Toronto – Scarborough, Toronto, ON Canada; 12 Department of Physiology, University of Toronto, Toronto, ON Canada

**Keywords:** Breastfeeding, DNA methylation, microRNA, BMI, Obesity, Mediation, ALSPAC, Child cohort, DOHaD

## Abstract

**Background:**

The role of breastfeeding in modulating epigenetic factors has been suggested as a possible mechanism conferring its benefits on child development but it lacks evidence. Using extensive DNA methylation data from the ALSPAC child cohort, we characterized the genome-wide landscape of DNA methylation variations associated with the duration of exclusive breastfeeding and assessed whether these variations mediate the association between exclusive breastfeeding and BMI over different epochs of child growth.

**Results:**

Exclusive breastfeeding elicits more substantial DNA methylation variations during infancy than at other periods of child growth. At the genome-wide level, 13 CpG sites in girls (*miR-21, SNAPC3, ATP6V0A1*, *DHX15/PPARGC1A*, *LINC00398/ALOX5AP*, *FAM238C*, *NATP/NAT2*, *CUX1*, *TRAPPC9*, *OSBPL1A*, *ZNF185*, *FAM84A*, *PDPK1*) and 2 CpG sites in boys (*IL16* and *NREP*), mediate the association between exclusive breastfeeding and longitudinal BMI. We found enrichment of CpG sites located within miRNAs and key pathways (AMPK signaling pathway, insulin signaling pathway, endocytosis). Overall DNA methylation variation corresponding to 3 to 5 months of exclusive breastfeeding was associated with slower BMI growth the first 6 years of life compared to no breastfeeding and in a dose–response manner with exclusive breastfeeding duration.

**Conclusions:**

Our study confirmed the early postnatal period as a critical developmental period associated with substantial DNA methylation variations, which in turn could mitigate the development of overweight and obesity from infancy to early childhood. Since an accelerated growth during these developmental periods has been linked to the development of sustained obesity later in life, exclusive breastfeeding could have a major role in preventing the risks of overweight/obesity and children and adults through DNA methylation mechanisms occurring early in life.

**Supplementary Information:**

The online version contains supplementary material available at 10.1186/s13148-021-01209-z.

## Background

It is now well established that early life exposure can impact our long-term health, in particular the risk of developing adult diseases such as obesity [1–6]. Nutritional factors affecting mothers before and during pregnancy can have profound and long-lasting consequences for the proper development of the fetus, which is known as “fetal programming” [7, 8]. After birth, nutritional factors during early infancy are critical to define the optimal growth, development, and future health of the individual later in life [7, 8]. Defective fetal programming may lead to permanent alterations resulting in a higher risk of obesity in childhood and other non-communicable chronic diseases, as well as later in life, termed metabolic programming [9, 10]. Conversely, postnatal intervention such as breastfeeding has the potential to mitigate risk factors and prevent metabolic and immune-related diseases.

The WHO suggests breastfeeding is the “perfect food for the newborn” and recommends all infants be exclusively breastfed up to 6 months of age, with continued breastfeeding along with appropriate complementary foods up to two years of age or beyond [11]. Importantly, there is growing evidence that breastfeeding may reduce the risk of being overweight [12, 13].

Specific nutrients and maternal antibodies found in human breast milk may explain some of the short-term protective effects of breastfeeding [14], but the mechanisms underlying its impacts on health throughout childhood, and extending into adulthood, remain largely unknown. There is also growing evidence that epigenetic factors play a major role in early life development [14–16] and might mediate the beneficial impacts of breastfeeding on child development. The most well understood epigenetic modification is DNA methylation (DNAm), which in mammals involves the addition of a methyl (-CH_3_) group to DNA at the 5' position of a cytosine base, typically at CpG dinucleotides. DNAm modifications have been correlated with intra-uterine growth, gestational age, birth weight, accelerated postnatal growth and can be triggered by dietary factors [17]. But the epigenetic signature related to breastfeeding remains largely unexplored. A candidate gene study suggested a negative association between the duration of breastfeeding and methylation level in blood cells in the promoter of the leptin (*LEP*) gene; a hormone that regulates energy homeostasis [15]. A recent study on exclusive breastfeeding (EBF) confirmed these findings in childhood (at 10 years) but not in young adulthood (at 18 years) [18]. The duration of breastfeeding has also been correlated to DNAm modifications in blood samples involving pathways such as cell signaling systems, development of anatomical structures and cells, development and function of the immune and central nervous systems [19]. A long-lasting effect of the duration of breastfeeding on DNA methylation at the *IL4R* (Interleukin-4 receptor) gene locus has been suggested at age 18 [20]. In a more recent epigenome-wise association study (EWAS), breastfeeding was associated with variations in the *TTC34* gene at age 7 and were still observed in adolescence [21]. Besides these findings, results on the role of breastfeeding as mediating important epigenetic pathways remain scarce. Previous studies have suffered a number of limitations including their small sample sizes, assessment of DNAm variations at a single time point rather than longitudinally and finally, their difficulty in establishing a causal relationship between breastfeeding, DNAm (as a mediator) and adiposity traits.

Our goals were thus to (a) Investigate the genome-wide landscape of blood DNAm variations in childhood associated with EBF and characterize its age- and sex-specific patterns; (b) Assess formally the mediation effect of childhood blood DNAm on the association between EBF and child BMI at different epochs of child growth and for varying EBF durations; c) Elucidate the different pathways and key epigenetic mechanisms contributing to this mediation. An overall representation of our overall conceptual framework is given in Additional file 1: Fig. S1.

## Results

### Characteristics of the study samples

In ALSPAC, a total of 358 boys and 374 girls with DNA methylation data and epidemiological information were available for our analysis. Children were included in the analysis if they had DNAm data available for at least two time points so that their longitudinal methylation profile could be estimated. The distribution of DNA samples by age and sex is given in Table 1. We found a higher proportion of girls vs. boys with EBF ≥ 3 months, 50.5% vs. 44.8% (Table 1). Other than that, the distribution of confounding variables is similar in boys and girls (Table 1).

**Table 1:** Distribution of the main epidemiological variables in ALSPAC

	Boys	Girls		Boys	Girls
	N = 358	N = 374		With DNA methylation available	
*EXBF (in months)*			*Age (years)*		
0	117 (32.7%)	116 (31.0%)	0	331	337
1	19 (5.3%)	24 (6.4%)	1	0	0
2	62 (17.3%)	45 (12.0%)	2	0	0
3	108 (30.2%)	125 (33.4%)	3	0	0
4	50 (14.0%)	47 (12.6%)	4	0	0
≥ 5	2 (0.6%)	17 (4.5%)	5	0	0
*Family weekly disposable income*			6	0	2
< £100	8 (2.2%)	6 (1.6%)	7	452	456
£1–199	43 (12.0%)	36 (9.6%)	8	0	0
£200–299	89 (24.9%)	103 (27.5%)	9	0	0
£300–399	88 (24.6%)	87 (23.3%)	10	0	0
> £400	116 (32.4%)	118 (31.1%)	11	0	0
Missing	14 (3.9%)	24 (6.4%)			
*Mother's education*			12	0	0
CSE/none	24 (6.7%)	27 (7.2%)	13	0	0
Vocational	28 (7.8%)	17 (4.5%)	14	3	1
O level	109 (30.4%)	126 (33.7%)	15	156	178
A level	112 (31.3%)	115 (30.7%)	16	196	186
University degree	85 (23.7%)	89 (23.8%)	17–18	83	104
*Mother's ever smoking status*					
No	319 (89.1%)	331 (88.5%)			
Yes	39 (10.9%)	43 (11.5%)			
*Birth weight (in kg)*					
Mean (± SD)	3.5 (± 0.5)	3.4 (± 0.4)			
*Mother's pre-pregnancy BMI*					
Mean (± SD)	22.9 (± 4.0)	22.8 (± 3.6)			
*Gestational age at birth (in weeks)*					
Mean (± SD)	39.6 (± 1.5)	39.7 (± 1.4)			

### DNA methylation profiles associated with EBF duration

**Fig. 1:**
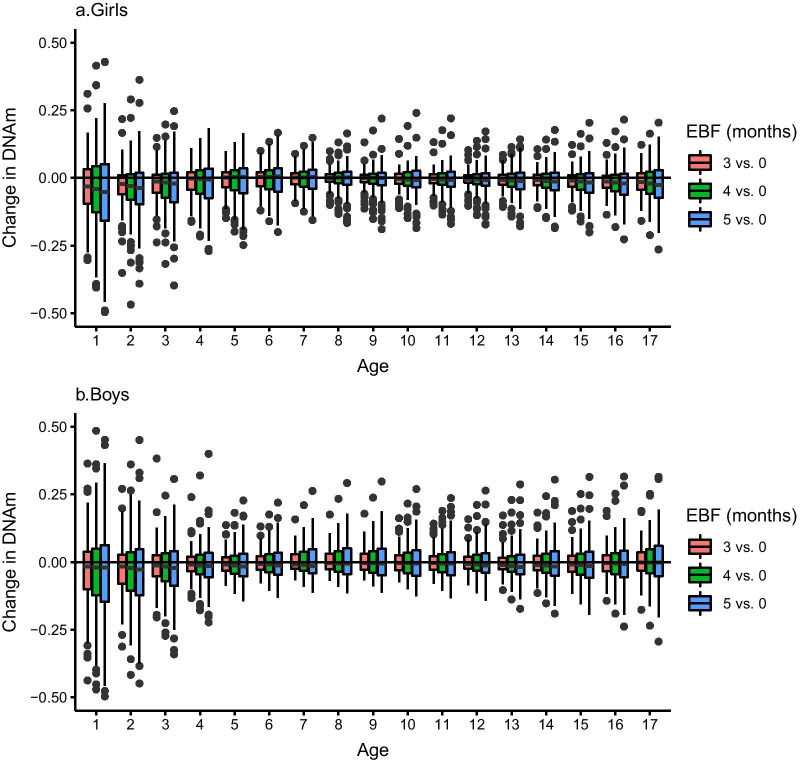
Distribution of DNAm variations induced by different durations (3,4 and 5 months vs. no breastfeeding) of exclusive breastfeeding (EBF) in ALSPAC girls (**a**) and boys (**b**) and at different ages from birth to 17 years. Each box represents the inter-quartile range (IQR) of DNAm variations over all CpG sites significant at *P* < 0.0001 (list given in Additional file 1: Table S1 for girls and Additional file 1: Table S2 for boys). The whiskers correspond to ± 1.5 IQR and the points outside this range are outliers

The profiles of DNAm variations associated with different durations of EBF are represented for the top 105 and 88 CpG sites, in girls and boys respectively, significant at the 10^−4^ significance level (Fig. 1). EBF is associated with much larger DNAm variations the first 3 years of life compared to other ages and in a dose-dependent manner with EBF duration. In ALSPAC girls, the average effect of 3 to 5 months EBF vs. no EBF is associated with DNAm variations (across all CpG sites) ranging from − 0.34 to 0.19 (M-value) the first year of life compared to − 0.23 to 0.07, − 0.09 to 0.06 and − 0.12 to 0.09 at age 2, 7 and 15 years, respectively (Additional file 1: Table S1). The corresponding DNAm variations in ALSPAC boys are − 0.49 to 0.88, − 0.35 to 0.64, − 0.08 to 0.18 and − 0.21 to 0.21 at age 1, 2, 7 and 15 years (Additional file 1: Table S2).

### EWAS hits identified at the genome-wide level

We found 13 and 2 CpG sites in girls and boys, respectively, reaching a genome-wide significance level of *P* < 5 × 10^−7^ (Table 2). The top two CpG sites are both associated with girls’ BMI (i.e., cg06471491 and cg16645539) and are located within the miRNA *miR-21* (*P* < 10^−16^) and the gene *SNAPC3* (*P* < 10^−16^). The average effect of 3 to 5 months EBF vs. no EBF is associated with an increase in the M-value of the *miR-21* CpG site by 0.02 one year postnatally, which mediates a BMI increase of 0.36 kg/m^2^. The same average effect of EBF is associated with a decrease in the M-value of the *SNAPC3* CpG site by 0.05 one year postnatally, which mediates a decrease in BMI of 0.38 kg/m^2^. The other top CpG sites are located within the following genes or genomic regions: *ATP6V0A1*, *DHX15/PPARGC1A*, *LINC00398/ALOX5AP*, *FAM238C*, *NATP/NAT2*, *CUX1*, *TRAPPC9*, *OSBPL1A*, *ZNF185*, *FAM84A*, *PDPK1* in girls, and *IL16* and *NREP* in boys.

**Table 2:** Top EWAS hits associated with child longitudinal BMI from ALSPAC data in boys and girls selected at the *P* = 5.10^−7^ level

CpG site	Gene	Chr	Pos. (kb)	Group	*P* value	EBF effect on DNAm (≥ 3 months vs. 0)^a^				EBF direct effect on BMI (≥ 3 months vs. 0)^b^				EBF effect on BMI mediated by DNAm (≥ 3 months vs. 0)^c^			
						1 year	2 years	7 years	15 years	1 year	2 years	7 years	15 years	1 year	2 years	7 years	15 years
*Girls*																	
cg06471491	miR-21	17	57,918	TSS1500	< E−16	0.02	− 0.08	− 0.07	− 0.07	− 1.13	− 0.51	− 0.17	− 0.37	0.36	0.11	0.00	0.01
cg16645539	SNAPC3	9	15,423	TSS200	< E−16	− 0.05	− 0.03	0.00	− 0.01	− 0.28	− 0.73	− 0.21	− 0.29	− 0.38	0.00	0.02	0.00
cg07408552	ATP6V0A1	17	40,612	5'UTR	3.E−08	− 0.19	− 0.18	0.06	0.00	− 1.84	− 0.44	− 0.16	− 0.32	− 0.34	− 0.08	0.03	− 0.01
cg08224066	DHX15/PPARGC1A	4	24,578	Body	4.E−08	− 0.04	0.03	0.05	0.05	− 0.30	− 0.44	− 0.20	− 0.30	0.00	0.03	0.00	0.02
cg14822546	LINC00398/ALOX5AP	13	31,381	–	7.E−08	− 0.01	0.01	0.01	− 0.01	− 0.76	− 0.53	− 0.22	− 0.40	0.09	− 0.04	0.01	0.00
cg14776321	FAM238C	10	27,220	Body	7.E−08	− 0.05	− 0.01	0.04	0.04	− 0.10	− 0.43	− 0.12	− 0.30	− 0.30	0.03	0.06	0.02
ch.8.18261148F	NATP/NAT2	8	18,217	–	7.E−08	− 0.11	− 0.06	− 0.03	− 0.04	− 0.66	− 0.67	− 0.19	− 0.26	0.05	0.01	0.00	0.01
cg00313685	CUX1	7	101,469	Body	1.E−07	0.19	0.06	− 0.01	0.05	− 0.39	− 0.14	− 0.22	− 0.30	− 0.10	0.00	− 0.02	− 0.04
cg17476951	TRAPPC9	8	141,054	Body	2.E−07	− 0.12	− 0.06	0.02	0.01	− 0.67	− 0.53	− 0.25	− 0.30	0.13	0.20	0.01	0.00
cg26398656	OSBPL1A	18	21,852	Body TSS200	3.E−07	− 0.11	− 0.06	0.00	0.04	− 0.36	− 0.60	− 0.23	− 0.25	− 0.27	− 0.11	0.03	− 0.01
cg26872564	ZNF185	X	152,087	Body	3.E−07	− 0.06	− 0.01	0.06	0.07	− 0.27	− 0.19	− 0.23	− 0.38	0.04	0.01	0.00	− 0.01
cg18176842	FAM84A	2	14,776	3'UTR	3.E−07	− 0.11	− 0.09	− 0.02	− 0.06	− 0.17	− 0.03	− 0.27	− 0.41	0.10	0.08	0.00	− 0.03
cg04354689	PDPK1	16	2,660	Body	5.E−07	0.19	0.06	− 0.01	0.05	− 0.39	− 0.14	− 0.22	− 0.30	0.14	0.02	− 0.02	0.00
*Boys*																	
cg26657240	IL16	15	81,475	–	3.E−07	− 0.02	− 0.02	− 0.03	− 0.03	− 1.43	− 0.39	− 0.01	− 0.06	− 0.01	0.00	0.05	0.23
cg08651538	NREP	5	111,070	Body	4.E−07	− 0.06	− 0.05	− 0.03	0.02	− 1.32	− 0.42	0.02	− 0.07	0.03	0.02	0.00	0.09

^a^Corresponds to estimate of β_M_(t) in Fig. 1. We estimated the average effect of 3 to 5 months of EBF vs. 0 month

^b^Corresponds to estimate of β_DIR_(t) in Fig. 1. We estimated the average effect of 3 to 5 months of EBF vs. 0 month

^c^Corresponds to estimate of β_IND_(t) in Fig. 1. We estimated the average effect of 3 to 5 months of EBF vs. 0 month

### Child BMI profiles associated with EBF and mediated by DNAm

**Fig. 2:**
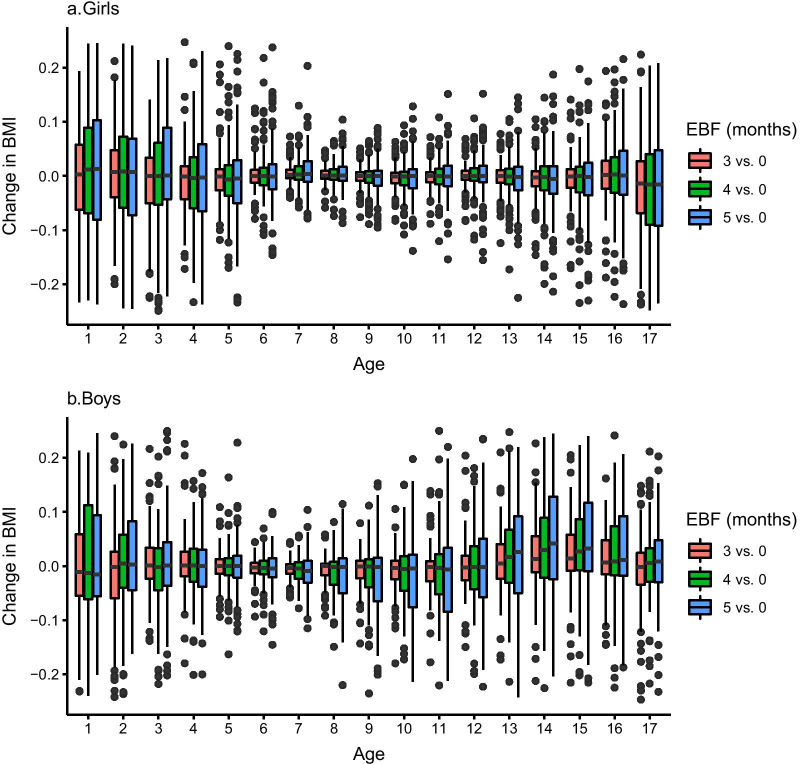
Distribution of mediation effects of DNA methylation on the association between different durations (3,4 and 5 months vs. no breastfeeding) of exclusive breastfeeding (EBF) and BMI in ALSPAC girls (**a**) and boys (**b**) and at different ages from birth to 17 years. Each box represents the inter-quartile range (IQR) of mediation effects over all CpG sites significant at *P* < 0.0001 (list given in Additional file 1: Table S1 for girls and Additional file 1: Table S2 for boys). The whiskers correspond to ± 1.5 IQR and the points outside this range are outliers

**Fig. 3:**
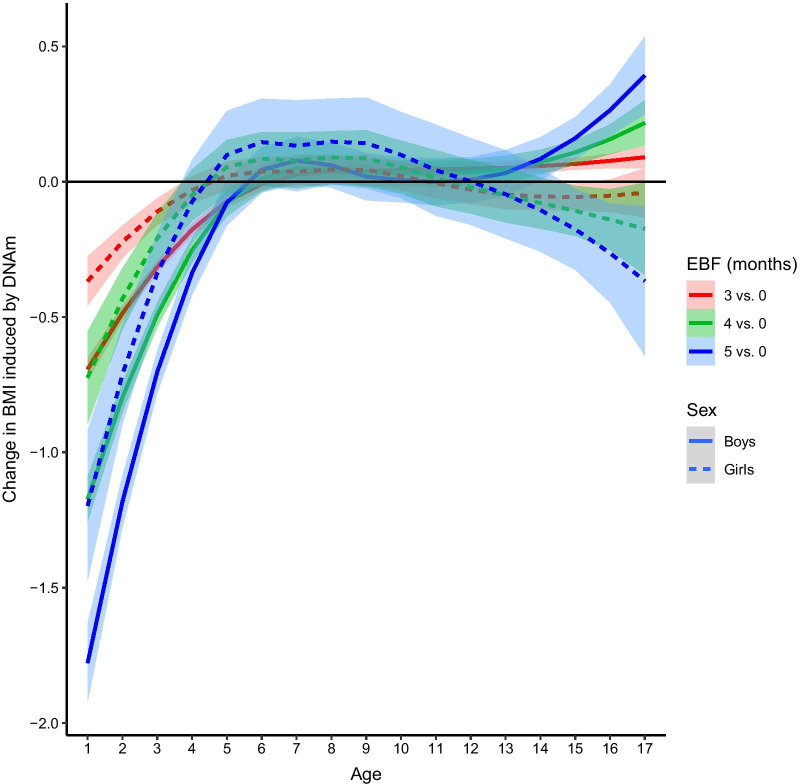
Change in BMI mediated by DNA methylation from the combined effect of multiple CpG sites where DNA methylation is induced by different durations (3,4 and 5 months vs. no breastfeeding) of exclusive breastfeeding (EBF) in ALSPAC girls and boys and at different ages from birth to 17 years. The CpG sites in the final multi-mediator model were selected by forward selection (see “Methods” section). Each curve represents a smooth estimate function (loess estimate) of BMI changes over time and the confidence bands are the 95% confidence intervals of the smoothed curve

The distribution of DNAm mediation effects (see “Methods” section) assessed over all CpG sites significant at the 10^−4^ significance level, has a similar pattern in girls and boys (Fig. 2). Larger variations are observed in the first 3 years of life compared to other ages and in a dose-dependent manner with EBF duration. We also assessed an overall mediation effect of CpG sites selected in a multi-mediator model on the association between EBF and BMI (see “Methods” section, Additional file 1: Table S3). Overall DNAm variation corresponding to 3 to 5 months EBF is associated with slower BMI growth the first 6 years of life compared to no EBF and in a dose–response manner with EBF duration (Fig. 3). This overall mediation effect then disappears after 6 years of age in both girls and boys (Fig. 3). A duration of 3 to 5 months EBF mediates 77% to 86% (1st year) and 65% to 80% (2nd year) of BMI changes in ALSPAC girls, and 86% to 91% and 92% to 94%, respectively, of BMI changes in ALSPAC boys.

### Functional annotation

We conducted a functional analysis of the top CpG sites significant at the 1 × 10^−4^ level with the software DAVID [22]. For girls, the top KEGG biological pathways include the “AMPK signaling pathway” (*P* = 0.034), the “Insulin signaling pathway” (*P* = 0.046), and “Endocytosis” (*P* = 0.050); and the top GO terms entail the ATP binding (*P* = 0.019) and multicellular organism development (*P* = 0.049) (Additional file 1: Table S4). In boys, “Pathways in cancer” is the only significant KEGG biological pathway at the 5% level and the top GO terms include “Negative regulation of transcription DNA-templated" (*P* = 1 × 10^−4^), “Positive regulation of tyrosine phosphorylation of Stat3 protein” (*P* = 0.020), “Negative regulation of transcription from RNA polymerase II promoter” (*P* = 0.031), “Regulation of cell shape” (*P* = 0.035), and “Hemopoiesis” (*P* = 0.036).

### CpG sites located in miRNAs

We found significant enrichment of CpG sites located within miRNAs among the top CpG sites selected at the 10^−4^ level, i.e., 4/113 in girls (*P* = 9.7 × 10^−3^, Fisher’s exact test) and 4/93 in boys (*P* = 5.1 × 10^−3^) for 3439 out of 485,512 CpG sites annotated to miRNAs. Among the 8 CpG sites significant at the 10^−4^ level (Additional file 1: Table S5), 6 of them have been previously found in breast milk (liqDB database). EBF from 3 to 5 months leads to lower methylation of 6 out of 8 of the associated miRNA CpG sites. The genomic landscape analysis indicates that the EWAS-significant CpG cg06471491 in *miR-21* (top CpG site in ALSPAC girls) is located approximately 500 bp upstream of *miR-21* (Additional file 1: Fig. S2). This region contains binding sites for multiple transcription factors and a TSS in the lymphoblastoid cell lines and embryonic stem cells. It is also included within DNAase hypersensitive sites, indicating some possible regulatory function. Interestingly, this CpG site is also significantly correlated with cg02782634 (Pearson *r* = 0.31, *P*-value < 1.10^−15^) and cg01409343 (Pearson *r* = 0.17, *P*-value < 1.10^−6^) in cord blood, which span an intronic region of the *VMP1* gene previously shown to include a promoter region for *miR-21* [23]. There is however uncertainty about the promoter regions of *miR-21* [23]. The genomic landscape analysis CpG site cg07143733 in *miR-155HG* (top CpG site in ALSPAC boys) shows that it is contained in a TSS region of the gene (Additional file 1: Fig. S3).

Finally, we also built gene–gene and gene-miRNA interaction networks from genes and miRNAs that had significant CpG sites at the 10^−4^ level, using the software OmicsNet [24] (Additional file 1: Fig. S4). It suggests a high inter-connectivity between the miRNA network and gene network.

### Underlying genetic variants

Among the top 15 EWAS hits, 8 are associated in *cis-* or *trans-* with an underlying genetic variant or with one or several genomic regions based on the mQTLdb database [25] (Additional file 1: Table S6). Among the 206 CpG sites significant at the 10^−4^ level (girls and boys combined), 74 are located in genes previously associated with BMI-related traits through genome-wide significant SNPs (Additional file 1: Tables S1 and S2). Adjusting our analyses for a 94-SNPs BMI-related polygenic risk score that we previously derived [26] did not change the significance of the individual CpG sites (Additional file 1: Tables S1 and S2) nor the overall mediation effect of all these CpG sites (Additional file 1: Fig. S5).

### Potential heath impact—example of the AMPK pathway

**Fig. 4:**
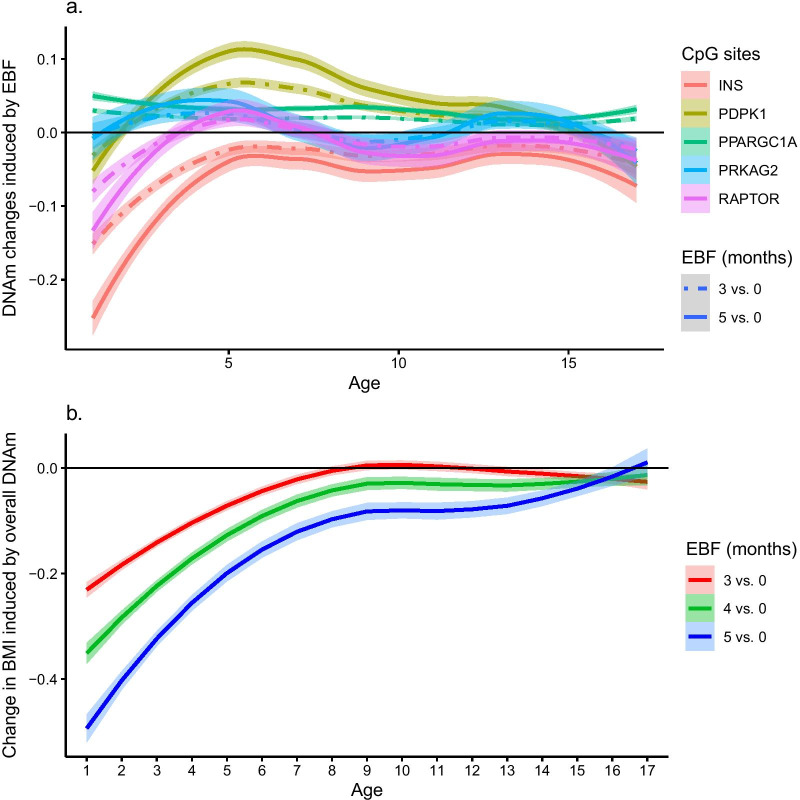
**a** Change in DNA methylation induced by different durations (3 and 5 months vs. no breastfeeding) of exclusive breastfeeding (EBF) in ALSPAC girls at different ages from birth to 17 years at 5 CpG sites linked to genes in the AMPK pathway. Each curve represents a smooth estimate function (loess estimate) of DNAm variations over time and the confidence bands are the 95% confidence intervals of the smoothed curve. **b** Change in BMI mediated by DNA methylation at 5 CpG sites linked to genes in the AMPK pathway where DNA methylation is induced by different durations (3,4 and 5 months vs. no breastfeeding) of exclusive breastfeeding (EBF) in ALSPAC girls at different ages from birth to 17 years. Each curve represents a smooth estimate function (loess estimate) of BMI changes over time and the confidence bands are the 95% confidence intervals of the smoothed curve

To illustrate the potential clinical implications of our findings, we consider the AMP-activated protein kinase (AMPK) pathway, which ranked first among the KEGG pathways discovered in ALSPAC girls. Interventions targeting DNAm variations of AMPK-related genes have already been suggested, in particular physical activity and bariatric surgery, with the aim of improving metabolic profiles and reducing obesity risks in the population [27]. In ALSPAC girls, EBF duration has a large impact on DNAm variations at 5 AMPK-related CpG sites that we found significant (Fig. 4a) and in a dose-dependent manner with EBF duration. The overall mediation effect of these 5 CpG sites is quite substantial across the first 8 years of life (Fig. 4b). A duration from 3 to 5 months EBF mediates from 62 to 68% and 71% to 75% of BMI changes across the first 2 years of life in ALSPAC girls. It suggests that an EBF-based intervention could mitigate the methylation of the AMPK pathway and impacts future child growth. Prolonged EBF-based interventions could be proposed to children who have a defective AMPK pathway, for example children from obese mothers.

### Replication

Out of 170 CpG sites we considered for replication in the Gen3G study, 2 probes passed the significance level of *P* < 2.9 × 10^−4^ (Additional file 1: Table S8) and were found only in girls: cg22879191 in the gene *CUX1* and cg19769982 in the miRNA *miR-155HG*. The CpG site cg22879191 in *CUX1* is significantly and negatively correlated with the other CpG site in this gene found in ALSPAC, cg00313685 (Pearson *r* = − 0.11, *P* = 5.2 × 10^−4^ in ALSPAC cord blood). The CpG site cg19769982 is located within a TSS of *miR-155HG* (Additional file 1: Fig. S3). It is significantly and negatively correlated with the other CpG site cg07143733 that we discovered in ALSPAC (Pearson *r* = − 0.13, *P* = 0.0001 in ALSPAC cord blood), possibly due to different regulatory activities of these two CpG sites. The pattern of histone modifications, location within DNase hypersensitive sites and the presence of binding sites for many transcription factors suggest that DNAm variations at the cg19769982 CpG site may affect the transcription of *miR-155HG*.

## Discussion

Evidence is mounting on the benefits of breastfeeding on human health, yet, little is known about the underlying biological mechanisms that confer this benefit. While the role of breastfeeding on DNAm has been long postulated, human studies assessing this role have been scarce and rather limited in scope. Our study brings new insights and extends previous knowledge by examining genome-wide DNAm variations at various epochs of child growth, in boys and girls, and in relation to the duration of EBF. Using one of the largest child cohorts worldwide, the ALSPAC study, we were able to comprehend (a) the full landscape of DNAm variations associated with EBF; (b) the role of DNAm as important mediator of the association between EBF and child BMI; (c) pathways and key epigenetic mechanisms underlying the role of EBF.

One of our major findings is that EBF elicits more substantial DNAm variations during infancy, including both more extreme hypo- and hyper-methylated CpG sites, than at other periods of child growth. The first 1000 days of an individual's life has been long recognized as the most critical period that shapes the development of future health outcomes and diseases such as obesity and also the most responsive to interventions [28]. The early postnatal life is also a period characterized by its particular epigenetic plasticity [29], although this has not been well investigated in human studies. Therefore, our study gives further evidence on the central role of EBF in regulating DNAm variations early in life. Besides, the alteration of DNAm variations was associated in a dose-dependent manner with the duration of EBF. We also investigated the role of non-exclusive breastfeeding and used EBF as a binary variable (less vs. more than 3 months), which did not change our main results, in particular the most significant CpG sites identified in our study. Our results also indicate sex-specific CpG sites/genes associated with EBF, which warrant further validation.

EBF elicits more substantial DNAm variations during infancy than at other periods of child development, which in turn mediate larger changes in BMI and in a dose-dependent manner with the duration of EBF from infancy to early childhood. Interestingly, EBF is associated with DNAm variations whose combined effects (overall mediation effect) promote slower BMI growth the first 6 years of life, in girls and boys, and this mediation effect decreases after this age. An accelerated growth during infancy has been clearly linked the development of sustained obesity later in life [30]. The role of EBF could therefore be to mitigate the risks of overweight/obesity through DNAm mechanisms occurring early in life. While a number of CpG sites were linked to underlying genetic variants (*cis*- or *trans*-mQTL), the mediation effect of those CpG sites persist even after adjustment for a BMI-related polygenic risk score. This is consistent with our previous analyses [26], which showed that this polygenic risk score is associated with increased child BMI but only after 6 years of age, whereas our present study suggests that DNAm variations mediate BMI growth before this age.

From a mechanistic standpoint, our study elucidated methylation genes/pathways most specifically targeted by EBF. A key mechanism emerging from our study involves DNAm variations at miRNA sites in child’s whole blood associated with EBF and which mediate child growth. This is supported by an overall enrichment of CpG sites located in several miRNAs, our top CpG site in girls lies within miR-21, which has been shown to be one of the most abundant miRNAs in breast milk [31, 32], and miR-155HG was replicated in the Gen3G study. This is also supported by the highly interconnected network of milk-related miRNAs and differentially methylated genes as a response to EBF in our study. While the role of breastmilk miRNAs as key epigenetic regulator of health and disease has been previously suggested [31, 32], this is the first time, to our knowledge, that their mediating effect is demonstrated and characterized over different epochs of child growth. Breast milk is a rich source of miRNAs and it has been suggested that milk miRNAs contained in exosomes could affect gene transcription and regulation of cellular events of the recipient's tissues, although evidence has been inconsistent [33]. For instance, Liao et al. [34] concluded that milk-derived miRNAs can be efficiently absorbed and regulate gene expression in a dose-dependent manner. Kusuma et al. [35] conducted experiments with human umbilical vein endothelium cells (HUVECs) and concluded that they can transport milk-derived exosomes by endocytosis. Interestingly, endocytosis is one of the top pathways mediating the role of EBF in our study and ATP binding (another of our top pathways) is needed to mediate this process. This latter study also observed the ability of milk exosomes to transfer across the endothelial layer as well as the compatibility of milk exosomes' surface proteins with the proteins on the surface of the HUVECs. The bio-availability of milk miRNAs in various tissues has also been demonstrated by several studies [36, 37]. Further, Wang et al. [38] confirmed with RNase-dependent PCR the presence of bovine milk-derived miRNAs in human plasma after its intake, especially miRNA-21 and miRNA-30a. Noteworthy, miRNA-21 is our top DNAm site mediating EBF. It is known to target mRNAs of important tumor suppressor proteins involved in upstream and downstream suppression of the rapamycin complex 1 (mTORC1) signaling pathway [39]. This nutrient-sensitive kinase pathway plays a critical role on the molecular level, as regulator of cell growth, cell proliferation, protein- and lipid synthesis, anabolic metabolic processes, and inhibition of autophagy [39]. The mTORC1 pathway is also activated by branched-chain amino acids, especially leucine, the most abundant amino acid of whey proteins, growth factors like insulin and insulin-like growth factor-1 (IGF-1), and sufficient cellular energy sensed by AMP-activated kinase (AMPK) [39]. Interestingly, AMPK is the top pathway identified in our study as mediator of EBF and the insulin signaling pathway, which plays a pivotal role in the regulation of energy homeostasis, was also outlined in our study. Therefore, the role of EBF could be to combine both amino acid- and miRNA-mediated pathways to optimize mTORC1 signaling for the promotion of postnatal growth.

Another mechanism identified by our study is the immune-regulatory effect of breast milk as outlined by other important pathways mediating EBF including the positive regulation of immune response, the chemokine-mediated signaling pathway and the cellular response to interleukin. It has also been suggested that immune-related miRNAs are abundant in breast milk exosomes [40] and could therefore partly mediate this immune-regulatory effect of breast milk. In ALSPAC boys, we identified a general KEGG pathway related to cancer and several GO terms related to the “Negative regulation of transcription” and to development mechanisms.

In terms of key genes, we were able to replicate the cut-like homeobox 1 (*CUX1*) gene, although a different probe was found in ALSPAC and Gen3G studies. It has previously been shown that *CUX1* could regulate the expression of the *FTO* and the *RPGRIP1L* genes and be involved in the coordination of the leptin receptor signaling pathway and its association with eating behavior [41]. *CUX1* and its related pathways such as the AMPK pathway could therefore be an important target of breastfeeding and might potentially play a role in modulating the risk of obesity in individuals carrying variants in the *FTO* gene [42].

Our study is based on one of the largest child cohorts worldwide with blood DNAm data and extensive epidemiological data available. We performed imputation of DNAm at all ages, assuming a linear trend between DNAm at any two time-points available. As our study demonstrates, the pattern of DNAm variations and BMI changes associated with EBF is highly age-specific and just performing cross-sectional analyses at specific ages might miss the fundamental role of breastfeeding on DNAm variations. We also provided a formal mediation analysis of DNAm as mediator of the association between EBF and child BMI in such a way that the role of DNAm cannot be confounded with other aspects of EBF directly associated with child BMI, e.g., nutrients present in breast milk which do not induce DNAm variations. Disentangling this mediation effect of DNAm is however challenging and would benefit from the collection of denser DNAm data around the early postnatal period. This remains a great challenge since the collection of biospecimens in infants may face important ethical barriers. Study of DNAm from whole blood might have limited the generalizability of our results since epigenetic mechanisms induced by EBF are likely to be tissue specific. Collecting DNA on multiple tissues would also be an invaluable resource for further validation of our results. Our analyses were adjusted for important epidemiological confounding factors, which is particularly important in mediation analyses, and potential biases such as batch effect and cell type heterogeneity.

## Conclusions

The outcomes of this research might have relevant health impacts. First, our study confirmed the early postnatal period as a critical developmental period. Our results suggest that substantial DNAm variations occur during this period. Second, this research has elucidated genes/pathways most specifically associated with EBF and determined a dose–response relationship between EBF duration, DNAm variations and their association with child growth. Such information could help design EBF-based interventions and select a possible target population most likely to benefit from it. As an example, we used the AMPK pathway, which has been advocated for targeted interventions such as physical activity and bariatric surgery [27] in order to prevent the risk of obesity. Our study suggests that an EBF-based intervention could mitigate the methylation of AMPK pathway and be associated with future child growth. Prolonged EBF-based interventions could even be proposed to children who have defective AMPK pathway, for example children from obese mothers. Finally, this work could help identify miRNAs responsible for breastfeeding-induced epigenetic variations, including those with positive impact and those detrimental to human health. As a whole, the evidence on milk miRNA bioactivity in health and disease remains very limited and warrants further investigations, both observational and experimental. If such function were to be validated, it could offer a tremendous paradigm shift, from a nutritional perspective, which would represent great opportunities for disease management [43, 44].

## Methods

### Study sample

Our discovery cohort is the Avon Longitudinal Study of Parents and Children (ALSPAC) [45, 46]. Pregnant women resident in Avon, UK with expected dates of delivery 1st April 1991 to 31st December 1992 were invited to take part in the study. The core ALSPAC sample consists of 14,541 pregnancies. An additional 542 eligible pregnancies not in the core sample, who were invited to participate at age 7 and for whom research data were available in November 2004, were included in this study. These 15,454 pregnancies resulted in 15,589 known fetuses of which 14,901 were alive at 1 year of age. For reasons of confidentiality data on the 13 triplet and quadruplet children were not available for analysis. After removing twins (*n* = 201) and children without anthropometric measures (height/length or weight) or age information (*n* = 2245), a total of 12,761 children were available for analysis. The children of these women have been followed for over two decades. Ethical approval for the study was obtained from the ALSPAC Ethics and Law Committee and the local research ethics committees. Informed consent for the use of data collected via questionnaires and clinics was obtained from all participants following the recommendations of the ALSPAC Ethics and Law Committee at the time. Please note that the study website contains details of all the data that is available through a fully searchable data dictionary and variable search tool [47].

### DNA methylation

In addition, 1018 ALSPAC mother–offspring pairs participated to the Accessible Resource for Integrated Epigenomics Studies (ARIES), which used both Illumina Infinium 450 k methylation arrays and Bisulfite-seq approaches to generate epigenetic data [48]. The ARIES participants were selected based on availability of DNA samples at two time points for the mother (antenatal and at follow-up when children were in adolescence) and at three time points for the infants/children (at birth, during childhood at age 7 and during adolescence at age 17). In this study, we only used the child methylation data. ARIES children provided a DNA sample extracted from cord blood drawn from the umbilical cord upon delivery (*n* = 1127 newborn babies). A second DNA sample was extracted from peripheral blood drawn at the 7-year clinic visit (*n* = 68 from white cells from buffy coats and *n* = 1018 from whole blood), and a third at a clinic held either at age 15 or 17 years, leading to three measurements of DNA methylation per child (*n* = 1073 from white cells from buffy coats on 1073). Pre-processing of the methylation data and normalization were performed by the ARIES team (see below). After pre-processing and accounting for missing epidemiological information, our dataset was restricted to 358 boys and 374 girls (Table 1). Beta-values, that measure the ratio of the methylated probe intensity over the overall intensity (sum of methylated and unmethylated probe intensities) [49], were derived on 481,166 probes. We transformed them into M-values to facilitate batch effect correction and the statistical analyses. To remove batch effects and adjust for cell type heterogeneity (i.e., whole blood vs. white blood cells), we used the Empirical Bayes method ComBat [50]. Finally, M-values were imputed between birth and age 7 and between age 7 and the third time-point available (between 15 and 17 years) based on simple linear regression.

### Pre-processing and analysis of the methylation data

The ARIES team [48] identified 369 technical replicates (361 duplicates + 8 triplicates), which were repeated in the lab due to poor quality samples (low detection rates). 112 samples failed the genotype quality controls (sample swaps, gender mismatches, high IBD or relatedness issues between mums and kids. 411 samples were removed due to mismatches using GWA concordance (concordance < 80%). 266 mismatches were identified using SNPprobes for concordance across family. 191 mismatches were identified using sex check (161 sex mismatches and 30 X–Y ratio outliers). 68 samples were identified as outliers when checking the methylated vs. unmethylated intensity. 14 samples were removed due to dye bias. The standard protocol by Illumina recommends excluding probes that have a detection p-value greater than an arbitrary cutoff of 0.05. The Aries team extracted detection p-values and detected 166 samples with a high proportion of undetected probes using a threshold of 0.01 (proportion of probes with *P*-value > 0.01 is > 0.1). The number of beads has also been extracted, 2 samples with a high proportion of probes with low bead number were removed (proportion of probes with bead number < 3 is > 0.1). Also 4246 probes were removed due to low bead numbers and low detection scores. The ARIES team then performed a functional normalization, which is a between-array normalization method for the Illumina Infinium HumanMethylation450 platform and an extension to quantile normalization. It removes unwanted technical variation by regressing out variability explained by the control probes present on the array. The normalization procedure is applied to methylated and unmethylated intensities separately, and to type I and type II signals separately. For the probes on the X and Y chromosomes, males and females are normalized separately using the sex information. For the Y chromosome, standard quantile normalization is used due to the small number of probes, which results in instability for functional normalization. Principal Component Analysis was used for post-normalization quality controls, 2 outliers were found and removed. ANOVA tests and t-tests were then performed to determine the association between the principal components and several batch variables (slide, plate, sample type, time code, time point), 11 samples were removed. A cell count normalization has also been performed for each sample individually based on a reference blood count dataset (gse35069 cell type methylation reference). The team noticed that it might be useful to remove the plate and slide effect since normalization did not efficiently removed this technical variation. All data pre-processing has been done using *R* package meffil [51]. After these pre-processing steps, a total of 615 outlier samples were removed. In addition, we excluded duplicated samples and some outliers defined based on ethnicity and genotype. The number of samples available for data analysis is indicated in Table 1.

### Transformation of the methylation data

A study demonstrated that beta-values have severe heteroscedasticity for highly methylated or unmethylated CpG sites [49]. The M-value provides much better performance in terms of Detection Rate (DR) and True Positive Rate (TPR) for both highly methylated and unmethylated CpG sites. We transformed the methylation beta-value of each CpG site into an M-value for our statistical analysis.

$$M-value={log}_{2}\left(\frac{\beta -value}{1-\beta -value}\right).$$

An M-value close to 0 indicates a similar intensity between the methylated and unmethylated probes, which means the CpG site is about half-methylated, assuming that the intensity data has been properly normalized [49]. Positive M-values mean that the CpG sites are more methylated than unmethylated, while negative M-values mean the opposite.

### Assessment of BMI

Birth length (crown-heel) was measured by ALSPAC staff who visited newborns soon after birth (median 1 day, range 1–14 days), using a Harpenden Neonatometer (Holtain Ltd). Birth weight was extracted from medical records. From birth to five years, length and weight measurements were extracted from health visitor records, which form part of standard childcare in the UK. In this cohort, there were up to four measurements taken on average at six weeks, 10, 21, and 48 months of age. For a random 10% of the cohort, length/height measurements from eight research clinic visits, held between the ages of four months and five years of age are also available. From age seven years upwards, all children were invited to approximately annual clinics. In addition, parent-reported child height and weight were also available from questionnaires. BMI was derived from height and weight measurements (mean 8 measures per person with a total of 45,534 measurements) and calculated as the weight (in kg) divided by the square of height (in m).

### Assessment of breastfeeding

Information pertaining to early infant feeding was collected. Mothers recorded the age at which breastfeeding was stopped (in months), and the age at which milk other than breast milk was introduced (in months). This information was determined from the mother’s diary of early feeding milestones, as well as from an interview with the study nurse at the 6 month child follow-up and survey questions at 15 month child follow-up. The duration of EBF was defined as the time from birth until feeding with other milk (non-breast milk) or any solid.

### Other epidemiological variables

Several known confounding variables were added to our multivariate analyses including mother’s pre-pregnancy BMI, gestational age (in months), total family income, mother's education level and pregnancy smoking status. The mother's BMI was obtained from the "About Yourself" questionnaire administered to the mother at 12 weeks gestation and calculated from weight and height measures. The gestational age was recorded in a variety of ways using last menstrual period, pediatric assessment, obstetric assessment and ultrasound assessment. The total family income measure was the weekly disposable income of the subject’s family at 33 months of age. The mother's education status was obtained from the "Your Pregnancy" questionnaire administered to the mother at 32 weeks gestation, it corresponds to the highest degree of education qualification and was coded as: Certificate of Secondary Education (CSE)/none; Vocational; O level; A level; University degree; Missing. The mother's pregnancy smoking status was obtained from "Having a Baby" questionnaire administered to the mother at 18 weeks gestation and was coded as: No/Yes. The variables education level and smoking status were analyzed as categorical variables while the variables gestational age, mother's BMI were analyzed as continuous variables.

### Epigenome-wide association study (EWAS) with linear mixed effect models (LMM)

The EWAS was performed separately in boys and girls using a mixed-effects model [52] with cubic spline basis. Longitudinal BMI from birth to 18 years of age was regressed on the CpG M-value, EBF duration, the CpG-by-EBF interaction, where those effects could vary with time according to a spline function sp(age).

Let ${y}_{i}=({y}_{i1}, {y}_{i2},\dots , {y}_{i{n}_{i}})$ denote the longitudinal BMI measurements for the *i*th individual with measurements at ages *t*_*ij*_ with *j* = *1,…,n*_*i*_. A child *i*’s BMI at age *t*_*ij*_ can be expressed in the general linear mixed-effects model framework as:

$${y}_{i}({t}_{ij})={X}_{i}^{T}({t}_{ij})\beta +{Z}_{i}^{T}{({t}_{ij})b}_{i}+{\varepsilon }_{i}({t}_{ij}), i=1,\dots ,N,$$

$${b}_{i}\sim N\left(0,G\right), { \varepsilon }_{i}\sim N\left(0, {\sigma }^{2}{R}_{i}\right),$$

where *β* is a vector of fixed effects, ${b}_{i}$ is a vector of random effects. ${X}_{i}$ and ${Z}_{i}$ are known fixed effects and random-effects regressor matrices which can include time-dependent variables (i.e., variables measured at age *t*_*ij*_), and ${\varepsilon }_{i}$ is the ${n}_{i}$-dimensional within-group error vector with a spherical Gaussian distribution. The random-effects regressor matrix ${Z}_{i}$ is a subset of the fixed-effects regressor matrix ${X}_{i}$. The parameter of *G* is the variance of random effects. And ${R}_{i}$ is first-order autoregressive correlation structure for continuous time variable for the dependence within-group errors ${\varepsilon }_{i}$ accounting for unbalance, unevenly spaced time points.

### Specific model for our analysis

For our analyses, we used this specific LMM:

1 $${y}_{i}\left({t}_{ij}\right)={X}_{i}^{T}\beta +{[W}_{i}^{T}\left({t}_{ij}\right){\beta }_{W}+{M}_{i}^{T}\left({t}_{ij}\right){\beta }_{M}+{W}_{i}^{T}\left({t}_{ij}\right) {.{M}_{i}^{T}\left({t}_{ij}\right){\beta }_{W.M}+ E}_{i}^{T}.{M}_{i}^{T}\left({t}_{ij}\right){\beta }_{E.M}].sp\left({t}_{ij}\right)+{ Z}_{i}^{T}{({t}_{ij})b}_{i}+{\varepsilon }_{i}({t}_{ij}),$$

where “.” represents the element-wise product, $sp\left({t}_{ij}\right)$ is a cubic spline function at age *t*_*ij*_ used to capture the nonlinear BMI growth curve and defined below, ${X}_{i}$ represents baseline confounding variables (maternal education, gestational age and the five most important principal components obtained from ALSPAC GWAS SNPs), and where *W*_*i*_(*t*_*ij*_), *M*_*i*_(*t*_*ij*_), *E*_*i*_*.M*_*i*_(*t*_*ij*_) correspond to time-varying confounding variables (mother’s BMI and tobacco use), methylation M-value, interaction between time-varying confounding variables and methylation and interaction between the duration of exclusive breastfeeding (in months) (EBF) and methylation at age *t*_*ij*_. Note that this model does not include an effect of EBF at baseline (i.e., at birth) since EBF is of course just introduced after birth. This LMM was fitted for cord blood and child blood DNAm at each single CpG site on the Illumina 450 K array.

We used cubic splines bases to specify sp(t) above to catch the peaks and valleys of the children's BMI growth trajectories without sharp corners. The cubic spline basis with k knots ${\mathcal{K}}_{j}, j=1,\dots ,k$ can be written as:

$$sp(t)=t+{t}^{2}+{t}^{3}+{\sum }_{j=1}^{k}{(t-{\mathcal{K}}_{j})}_{+}^{3}$$

where

$${(t-{\mathcal{K}}_{j})}_{+}=\left\{\begin{array}{c} t-{\mathcal{K}}_{j} \; if \;t> {\mathcal{K}}_{j} \\ 0 \; if \; t\le {\mathcal{K}}_{j} \end{array} j=1,\dots ,k.\right.$$

The likelihood ratio test (LRT) and Akaike Information Criterion (AIC) were used to select the optimal knots and the number of knots. The optimal knots were (0.7, 1.5, 10) for boys and (0.9, 1.5) for girls.

### Hypothesis testing

The General Linear Hypothesis (GLH) approach [53] was used for hypothesis testing. The GLH test is based on the normal approximation for maximum likelihood estimators using the estimated variance–covariance matrix. The hypothesis can be specified through a constant matrix *L* to be matched with the fixed effects of the model such that ${H}_{0}:L\beta =m$ where the *m* is the hypothesized value. The estimate of fixed effects $\beta$ follows asymptotically a multivariate normal distribution $\widehat{\beta }\sim N(\beta , cov\left(\widehat{\beta }\right))$ based on Central Limit Theorem such that the linear form $L\widehat{\beta }$ also follows asymptotically a multivariate normal distribution: $L\widehat{\beta }\sim N\left(L\beta , Lcov\left(\widehat{\beta }\right){L}^{^{\prime}}\right).$ Therefore, the *p*-value and the 95% confidence interval for the hypothesized value can be obtained accordingly. An ANOVA test was used to test each main component of the LMM in (1), which corresponds to testing the null hypothesis: ${H}_{0}:L\beta =0$ and where *L* is a vector of 1. Our main interest was to perform an ANOVA test for the components ${\beta }_{E.M}$ and ${\beta }_{M}$. The rationale was to identify CpG sites whose DNAm variations over time are associated with child growth $({\beta }_{M})$ and this association can be altered by EBF duration ${\beta }_{E.M}$. The resulting p-values are denoted *p*_*1*_ and *p*_*2*_ and we have $T=-2log({p}_{1})-2log({p}_{2})\sim {\chi }^{2}(4)$. The most significant CpG sites were selected based on this *T* test statistic at the level of *P* < 5 × 10^−7^, as suggested by a previous study [54], in boys and girls separately. We also considered a significance level of *P* = 1 × 10^−4^ to decide which CpG sites and their associated gene to include in our pathway-based analysis and for characterizing the overall DNA profiles and mediation effect profiles (see “Results” section and Figs. 1, 2, 3) associated with EBF. A genomic control approach [55] was used to correct a possible deviation from the asymptotic chi-square distribution (Additional file 1: Fig. S6).

### Main outcomes

Our outcomes of interest correspond to (1) DNAm variations (M-value) associated with 3 to 5 months of EBF compared to no breastfeeding; (2) BMI changes associated with 3 to 5 months of EBF compared to no breastfeeding mediated by DNAm (indirect effect of EBF on longitudinal BMI) and not mediated by EBF (direct effect of EBF on longitudinal BMI) (Table 2).

### Mediation analysis in longitudinal studies

We performed a mediation analysis to characterize the natural direct effect (NDE) and natural indirect effect (NIE) of EBF on the longitudinal BMI, where the indirect effect is modulated by DNA methylation using the approach of Bind et al. [56]. For these analyses, we calculated the predicted child-specific BMI trajectories by combining the estimated fixed effects, i.e., the population average shared by all individuals, with the child-specific predicted random effects up to age 18 years from the mixed model described above. We also predicted the methylation M-value conditional on the duration of EBF and other confounding covariates using the following LMM:

2 $${M}_{i}\left({t}_{ij}\right)={X}_{i}^{T}\gamma +{[W}_{i}^{T}\left({t}_{ij}\right){\gamma }_{W}+{E}_{i}^{T}{\gamma }_{E}].sp\left({t}_{ij}\right)+{ Z}_{i}^{T}{({t}_{ij})b^{\prime}}_{i}+{\varepsilon ^{\prime}}_{i}({t}_{ij}).$$

Following Bind et al. [56], the NDE at any time *t* between birth and 18 years of age is defined as:

3 $$NDE(t)=\left(\widehat{y}(t)|E=e, \widehat{(M}(t)|E=0)\right)-\left(\widehat{y}(t)|E=0, (\widehat{M}(t)|E=0)\right),$$

that is, the NDE contrasts at any time *t* the predicted BMI ($\widehat{y})$ estimated from model (1) corresponding to a level of EBF, *E* = *e* (between 1 to 5 months in our analyses) and where the methylation level is estimated at a level of EBF of 0 month from model (2).

The NIE at any time *t* between birth and 18 years of age is defined as:

4 $$NIE(t)=\left(\widehat{y}(t)|E=e, \widehat{(M}(t)|E=e)\right)-\left(\widehat{y}(t)|E=e, \widehat{(M}(t)|E=0)\right),$$

The NIE contrasts the predicted BMI ($\widehat{y})$ corresponding to a methylation estimated from model (2) for a level of EBF, *E* = *e* (between 1 to 5 months in our analyses) and a predicted BMI corresponding to a methylation estimated at *E* = 0. The effect of EBF on BMI corresponds is set at *E* = *e*. The total effect of EBF on BMI at any time point *t* is the sum of *NDE*(*t*) and *NIE*(*t*). The proportion of EBF effect on BMI mediated by methylation is simply given by *NIE*(*t*)/(*NDE*(*t*) + *NIE*(*t*)).

For these mediation analyses, all continuous confounding variables were set at 0 as well as the random effects (mean population value) and the confounding categorical variables were set at the most frequent category.

We also considered a multi-mediator model [56] where multiple CpG sites can mediate jointly the association between EBF and BMI. This model is useful to assess an “overall” DNAm mediation effect. We first fitted model (1) and applied a forward selection procedure to select which CpG site to enter the model among all CpG sites significant in univariate analyses at the 10^−4^ level. The probability for a new CpG site to enter the model was set at *P* = 10^−4^ and the probability to be withdrawn from the model at 10^−3^. The *NDE*(*t*) and *NIE*(*t*) values were then calculated following Eqs. (3) and (4) except that $\widehat{M}(t)|E=e$ is now replaced by $\widehat{M1}\left(t\right)\left|E=e+\dots +\widehat{Mp}\left(t\right)\right|E=e,$ where *p* is the number of CpG sites selected in the multivariate model and each $\widehat{Mj}\left(t\right)|E=e$ value is estimated separately from model (2). All the statistical analyses were performed with the statistical software *R* version 3.6.3.

### Functional annotation and enrichment analyses

We also examined CpG sites significant at the 5 × 10^−4^ level for a pathway-based analysis with the software DAVID [22]. The top enriched gene ontology (GO) terms and KEGG pathways were selected at the 5% significance level. To help understand possible regulatory roles of DNAm in biological pathways, we examined the genomic landscape of the EWAS-significant CpGs using the Ensembl Genome browser [57]. Finally, we built gene–gene and gene-miRNA interaction networks from our discovered genes and miRNAs using the software OmicsNet [24].

### Replication cohort and analyses

The Gen3G cohort [58] was used for the replication of the top CpG sites discovered in the ALSPAC study at the genome-wide level of *P* < 5.10^−7^. Our concept of replication in Gen3G was taken in the broader sense of any CpG site that was significantly correlated with the initial CpG site discovered in ALSPAC and located within the same gene or its promoter region. This correlation could be positive or negative and was assessed in ALSPAC cord blood. Gen3G is a prospective observational cohort which enrolled pregnant women age ≥ 18 years old, between January 2010 and June 2013, from the Centre Hospitalier Universitaire de Sherbrooke (CHUS) in the Estrie region of Quebec, Canada. At delivery, a total of 854 pregnancies were included in the study. Prospective follow-ups of mothers and children were performed at 3 and 5 years of age. DNAm data were obtained in children at 5 years of age, using the Infinium MethylationEPIC BeadChip Arrays (Illumina Inc, USA). Processing was performed including normalization and bias correction using funnorm [59] and RCP [60], respectively. After pre-processing and accounting for missing epidemiological information, the Gen3G dataset was restricted to 104 boys and 89 girls (Additional file 1: Table S7 for a description of the data). DNAm was analyzed as M-value. For replication, we have included all CpG sites from Gen3G present in our top genes (Table 2) and all the CpG sites present in the top miRNAs (Additional file 1: Table S5). This led to 170 CpG sites to replicate and we used 0.05/170 = 2.9 × 10^−4^ for the replication p-value. Similarly to our analyses in ALSPAC, we performed in Gen3G an ANOVA test for the components ${\beta }_{E.M}$ and ${\beta }_{M}$ from a simple regression model:

$${y}_{i}={X}_{i}^{T}\beta +{M}_{i}^{T}{\beta }_{M}{+ E}_{i}^{T}.{M}_{i}^{T}{\beta }_{E.M}+{\varepsilon }_{i},$$

The resulting p-values are denoted *p*_*1*_ and *p*_*2*_ and we have $T=-2log({p}_{1})-2log({p}_{2})\sim {\chi }^{2}(4)$. The confounding variables *X*_*i*_ include mother's education at the 5 years visit, mother's smoking status at first trimester of pregnancy (yes/no), mother's BMI at first trimester of pregnancy, gestational age at birth, child BMI at birth and child ethnic group (Caucasians vs. non-Caucasians) (Additional file 1: Table S7).

## Supplementary Information

**Additional file 1.** Supplementary tables and figures.

## Data Availability

Data from the ALSPAC cohort is available to researchers according to processes outlined at http://www.bristol.ac.uk/alspac/researchers/access/. Individual level data have restricted access and are distributed upon approval of research proposal and payment of data access costs.

## Abbreviations

ALSPAC: Avon Longitudinal Study of Parents and Children
AMPK: AMP-activated protein kinase
ARIES: Accessible Resource for Integrated Epigenomics Studies
BMI: Body Mass Index
CSE: Certificate of Secondary Education
DNAm: DNA methylation
DR: Detection rate
EBF: Exclusive breastfeeding
EWAS: Epigenome-wide association study
GLH: General linear hypothesis
GWA: Genome-wide association
IL4R: Interleukin-4
LEP: Leptin
LMM: Linear mixed effect models
mQTL: Methylation quantitative trait loci
NDE: Natural direct effect
NIE: Natural indirect effect
TPR: True positive rate
TSS: Transcription start site
WHO: World Health Organization
