# Supplementary material for: DNA methylation mediates the association between breastfeeding and early-life growth trajectories
Source: Clin Epigenetics. 2021 Dec 22;13:231. doi: 10.1186/s13148-021-01209-z (PMC8697471; doi:10.1186/s13148-021-01209-z)

Supplementary Table 1: Top EWAS hits from ALSPAC data in girls selected at the P=1.10^{-4} level.

**CpG site**	**Gene**	**Chr**	**Pos.**	**Group**	***GWAS SNP***	***P***	***P*adj†**	**EBF effect on DNAm (≥3 months vs. 0)#**				**EBF direct effect on BMI (≥3 months vs. 0)$**				**EBF effect on BMI mediated by DNAm (≥3 months vs. 0)&**			
								**1 year**	**2 years**	**7 years**	**15 years**	**1 year**	**2 years**	**7 years**	**15 years**	**1 year**	**2 years**	**7 years**	**15 years**
**cg06471491**	miR-21	17	57,918	TSS1500	N	<E-16	<E-16	0.02	-0.08	-0.07	-0.07	-1.13	-0.51	-0.17	-0.37	0.36	0.11	0.00	0.01
**cg16645539**	SNAPC3	9	15,423	TSS200	rs62571018 BMI	<E-16	<E-16	-0.05	-0.03	0.00	-0.01	-0.28	-0.73	-0.21	-0.29	-0.38	0.00	0.02	0.00
**cg07408552**	ATP6V0A1	17	40,612	5'UTR	rs11079041 Waist-hip ratio	3.E-08	2.E-08	-0.19	-0.18	0.06	0.00	-1.84	-0.44	-0.16	-0.32	-0.34	-0.08	0.03	-0.01
**cg08224066**	DHX15/ PPARGC1A	4	24,578	Body	N	4.E-08	2.E-08	-0.04	0.03	0.05	0.05	-0.30	-0.44	-0.20	-0.30	0.00	0.03	0.00	0.02
**cg14822546**	LINC00398/ ALOX5AP	13	31,381		N	7.E-08	8.E-08	-0.01	0.01	0.01	-0.01	-0.76	-0.53	-0.22	-0.40	0.09	-0.04	0.01	0.00
**cg14776321**	ANKRD26	10	27,220	Body	rs7081476 BMI	7.E-08	2.E-07	-0.05	-0.01	0.04	0.04	-0.10	-0.43	-0.12	-0.30	-0.30	0.03	0.06	0.02
**ch.8.18261148F**	NATP/NAT2	8	18,217		rs4921913 Metabolic syndrome	7.E-08	1.E-07	-0.11	-0.06	-0.03	-0.04	-0.66	-0.67	-0.19	-0.26	0.05	0.01	0.00	0.01
**cg00313685**	CUX1	7	101,469	Body	rs367026 Waist-hip ratio	2.E-07	2.E-07	0.19	0.06	-0.01	0.05	-0.39	-0.14	-0.22	-0.30	-0.10	0.00	-0.02	-0.04
**cg17476951**	TRAPPC9	8	141,054	Body	rs71514664 Hip circum ference	2.E-07	6.E-07	-0.12	-0.06	0.02	0.01	-0.67	-0.53	-0.25	-0.30	0.13	0.20	0.01	0.00
**cg26398656**	OSBPL1A	18	21,852	Body; TSS200	rs275856 Type 2 diabetes	3.E-07	5.E-07	-0.11	-0.06	0.00	0.04	-0.36	-0.60	-0.23	-0.25	-0.27	-0.11	0.03	-0.01
**cg26872564**	ZNF185	X	152,087	Body	N	3.E-07	2.E-06	-0.06	-0.01	0.06	0.07	-0.27	-0.19	-0.23	-0.38	0.04	0.01	0.00	-0.01
**cg18176842**	NSE1	2	14,776	3'UTR	rs7602441 BMI	3.E-07	4.E-07	-0.11	-0.09	-0.02	-0.06	-0.17	-0.03	-0.27	-0.41	0.10	0.08	0.00	-0.03
**cg04354689**	PDPK1	16	2,661	Body	N	5.E-07	6.E-07	0.19	0.06	-0.01	0.05	-0.39	-0.14	-0.22	-0.30	0.14	0.02	-0.02	0.00
**cg03664802**	EPC2	2	149,402	TSS200	rs1234423 Body height	5.E-07	5.E-07	0.05	0.00	-0.01	-0.04	-0.71	-0.37	-0.24	-0.34	-0.01	0.04	0.01	0.00
**cg04781580**	TRAV14DV4	14	22,393		N	6.E-07	3.E-07	-0.01	0.03	-0.02	-0.02	-0.31	-0.42	-0.22	-0.38	0.11	-0.01	0.03	0.06
**cg11746359**	MAML3	4	141,012	Body	rs2667360 BMI	6.E-07	4.E-07	-0.21	-0.11	0.01	-0.03	-0.52	-0.33	-0.22	-0.40	-0.09	0.02	0.01	0.01
**cg27215185**	GMDS	6	2,198	Body	rs143303199 Body height	1.E-06	2.E-06	-0.11	-0.06	0.00	0.04	-0.36	-0.60	-0.23	-0.25	-0.21	0.13	-0.01	0.00
**cg23057721**	LINC02594	17	41,791		N	1.E-06	2.E-06	-0.10	-0.04	0.04	-0.01	-0.61	-0.05	-0.31	-0.47	-0.09	0.07	0.02	0.02
**cg04738270**	ACTR1A	10	104,262	Body	rs3862030 Waist cir cumference	2.E-06	3.E-06	0.05	0.04	0.01	0.07	-0.71	-0.37	-0.27	-0.46	-0.53	-0.14	0.02	-0.01
**cg09029192**	TNRC6C	17	76,015	5'UTR	rs1656794 Body height	2.E-06	3.E-06	-0.05	-0.01	0.04	0.04	-0.10	-0.43	-0.12	-0.30	-0.05	0.00	0.00	-0.01
**cg11566521**	RAPGEF2	4	160,128		rs563875 Body height	2.E-06	7.E-07	-0.04	0.03	0.05	0.05	-0.30	-0.44	-0.20	-0.30	0.02	0.06	-0.01	-0.04
**cg00524694**	PROP1	5	177,388		rs6601211 Body height	3.E-06	3.E-06	-0.19	-0.18	0.06	0.00	-1.84	-0.44	-0.16	-0.32	-0.02	0.00	0.01	0.00
**cg00070899**	GRM4	6	34,024	Body	rs1759645 Body height	3.E-06	3.E-06	-0.21	-0.11	0.01	-0.03	-0.52	-0.33	-0.22	-0.40	0.17	0.06	0.00	-0.02
**cg23027583**	HLX	1	221,053	TSS200	rs10218741	3.E-06	5.E-06	0.05	0.04	0.01	0.07	-0.71	-0.37	-0.27	-0.46	0.01	-0.02	0.01	0.00

					Body height														
**cg05241732**	MYH6/ miR-208A	14	23,859	Body; TSS1500	rs2231301 Body height	3.E-06	1.E-05	-0.20	-0.12	0.03	0.00	-0.28	-0.22	-0.26	-0.39	-0.04	-0.02	-0.01	0.01
**cg26992566**	EMC8	16	85,814	Body	N	5.E-06	5.E-05	-0.12	-0.06	0.02	0.01	-0.67	-0.53	-0.25	-0.30	-0.06	-0.04	0.01	0.01
**cg23543615**	MEF2B	19	19,281	Body; 5'UTR	rs11670016 waist-hip ratio	5.E-06	9.E-06	-0.02	-0.02	0.01	-0.02	-0.40	-0.59	-0.20	-0.30	-0.15	-0.04	0.01	0.00
**cg01505676**	TGM7	15	43,595	TSS1500	N	6.E-06	8.E-06	-0.07	-0.03	0.00	0.02	-0.29	-0.45	-0.16	-0.30	-0.23	-0.06	0.02	0.00
**cg17862404**	TSC22D1	13	45,009	Body	rs10507524 waist-hip ratio	7.E-06	9.E-06	0.07	0.07	0.05	0.00	-0.35	-0.27	-0.20	-0.27	-0.59	-0.12	0.05	0.01
**cg06683602**	CXCR1	2	219,032	TSS1500	N	7.E-06	2.E-05	-0.20	-0.12	0.03	0.00	-0.28	-0.22	-0.26	-0.39	-0.88	-0.51	-0.05	0.00
**cg23662178**	ZNF518A	10	97,884		rs1172955 Body height	7.E-06	7.E-06	-0.03	-0.01	0.02	0.02	-0.46	-0.16	-0.25	-0.47	0.15	0.12	-0.01	0.01
**cg21966975**	PADI3	1	17,575	TSS200	rs2284746 Body height	8.E-06	6.E-06	-0.01	0.03	-0.02	-0.02	-0.31	-0.42	-0.22	-0.38	-0.20	-0.05	0.03	-0.14
**cg22851175**	DDX11	12	31,274		rs3214606 Type 2 diabetes	9.E-06	2.E-05	0.15	0.05	-0.09	-0.06	-0.52	0.12	-0.24	-0.35	-0.49	-0.19	-0.01	0.08
**cg14531663**	PRRT1	6	32,117	3'UTR	rs2857693 Body height	1.E-05	6.E-06	-0.19	-0.18	0.06	0.00	-1.84	-0.44	-0.16	-0.32	-0.29	0.24	0.03	0.00
**cg10053073**	RECQL5	17	73,623	3'UTR	rs2385263 Waist-hip ratio	1.E-05	3.E-05	0.07	0.07	0.05	0.00	-0.35	-0.27	-0.20	-0.27	-0.02	-0.01	0.00	-0.04
**cg07548512**	SDF4	1	1,155	Body	rs1240697 Body height	1.E-05	1.E-05	-0.15	-0.07	0.03	0.02	-0.29	-0.41	-0.17	-0.32	0.22	0.01	-0.01	-0.01
**cg17401282**	GABRG2	5	161,494	TSS1500	N	1.E-05	4.E-05	-0.05	-0.03	0.00	-0.01	-0.28	-0.73	-0.21	-0.29	0.18	0.21	-0.01	-0.04
**cg12510087**	GALNT9	12	132,887	Body	rs11247009 BMI	1.E-05	8.E-05	-0.06	-0.01	0.06	0.07	-0.27	-0.19	-0.23	-0.38	-0.10	-0.10	-0.02	0.16
**cg10082165**	CTSG	14	25,044	Body	N	2.E-05	2.E-05	-0.11	-0.04	0.04	-0.05	-0.28	0.05	-0.16	-0.33	-0.01	0.00	0.01	-0.03
**cg06340161**	GPT2	16	46,963	3'UTR	N	2.E-05	5.E-05	0.00	0.05	0.05	-0.01	0.40	-0.07	-0.24	-0.57	-0.06	0.02	0.01	-0.03
**cg12251803**	LRRFIP1	2	238,640	Body	rs11680012 obesity	2.E-05	3.E-05	-0.34	-0.23	-0.04	-0.12	-0.27	-0.27	-0.21	-0.40	-0.18	-0.26	-0.03	0.00
**cg22334632**	TMEM160	19	47,553	TSS1500	rs112551143 BMI	2.E-05	3.E-04	0.00	0.05	0.05	-0.01	0.40	-0.07	-0.24	-0.57	0.00	0.05	0.04	0.01
**cg09560549**	SPG7	16	89,573	TSS1500	rs4785574 Body height	2.E-05	6.E-05	-0.01	0.00	-0.01	-0.01	-0.79	-0.72	-0.21	-0.35	0.03	-0.18	0.00	-0.01
**cg04516203**	N/A	16	51,443		N	2.E-05	2.E-05	0.14	0.00	-0.07	-0.07	-0.97	-0.36	-0.19	-0.37	-0.12	-0.18	0.00	0.00
**cg03553386**	IGF2;INS-IGF2	11	2,171	TSS200; Body	rs217227 Body height	2.E-05	8.E-05	-0.10	-0.04	0.04	-0.01	-0.61	-0.05	-0.31	-0.47	-0.65	-0.21	-0.02	0.02
**cg14086922**	MICAL3	22	18,268		rs10854521 Body height	2.E-05	3.E-05	-0.04	-0.02	-0.01	0.01	-0.78	-0.89	-0.21	-0.28	-0.09	-0.18	-0.04	0.09
**cg19497523**	TMPRSS9	19	2,425	Body	N	3.E-05	8.E-06	-0.04	-0.02	-0.01	0.01	-0.78	-0.89	-0.21	-0.28	0.13	0.10	0.00	0.04
**cg03409272**	CCL14-CCL15	17	34,330	TSS1500	N	3.E-05	3.E-05	-0.21	-0.11	0.01	-0.03	-0.52	-0.33	-0.22	-0.40	-1.49	-0.52	0.02	0.04
**cg02822745**	DKK2	4	108,271		N	3.E-05	3.E-05	-0.01	0.00	-0.01	-0.01	-0.79	-0.72	-0.21	-0.35	0.75	0.37	0.00	-0.06
**cg16004911**	FRYL	4	48,783	TSS1500	rs10031777 Body height	3.E-05	5.E-05	0.02	-0.08	-0.07	-0.07	-1.13	-0.51	-0.17	-0.37	0.08	0.00	0.00	0.03
**cg07988060**	UTP6	17	30,235		rs7209766 Body height	3.E-05	2.E-05	-0.05	-0.03	0.00	-0.01	-0.28	-0.73	-0.21	-0.29	-0.01	0.06	0.01	-0.01
**cg23000550**	P2RX6	22	21,369	TSS1500	N	3.E-05	9.E-05	-0.16	-0.06	0.05	-0.12	-0.46	0.07	-0.40	-0.54	-0.44	-0.30	0.01	0.00
**cg15921911**	RAD51B	14	69,156		rs11158743 Body height	3.E-05	7.E-05	-0.34	-0.23	-0.04	-0.12	-0.27	-0.27	-0.21	-0.40	-0.62	-0.64	0.01	-0.06
**cg15243597**	WDPCP	2	63,553	Body	rs775417095 Waist cir cumference	3.E-05	4.E-05	-0.07	-0.03	0.00	0.02	-0.29	-0.45	-0.16	-0.30	0.11	0.17	0.01	0.00
**cg07364240**	NRROS	3	196,389	3'UTR	N	4.E-05	2.E-05	-0.15	-0.07	0.03	0.02	-0.29	-0.41	-0.17	-0.32	-0.12	-0.03	0.01	-0.08
**cg07012178**	PRKAG2	7	151,305	Body	N	4.E-05	5.E-05	0.00	0.05	0.05	-0.01	0.40	-0.07	-0.24	-0.57	-0.07	-0.13	0.01	-0.08
**cg10883421**	RMND1/	6	151,773	TSS200	rs9397403	4.E-05	2.E-05	0.02	-0.08	-0.07	-0.07	-1.13	-0.51	-0.17	-0.37	-0.55	0.14	0.06	-0.04

	ARMT1				Body height												
**cg06527213**	KLHL25/ MIR1276	15	86,315	5'UTR; TSS1500	rs10520587 Body height	4.E-05	7.E-05	-0.04	-0.03	-0.05	-0.05	-0.92	-1.03	-0.22	-0.31	-0.13	-0.03	-0.02	-0.09
**cg02754470**	RNF111	15	59,158		N	4.E-05	9.E-05	-0.11	-0.09	-0.02	-0.06	-0.17	-0.03	-0.27	-0.41	-0.05	-0.04	0.01	-0.03
**cg26144456**	NXT2	X	108,779	TSS200	N	4.E-05	2.E-05	-0.04	-0.03	-0.05	-0.05	-0.92	-1.03	-0.22	-0.31	0.15	0.07	0.01	0.02
**cg09699373**	USP10	16	84,823		rs2326458 Body height	4.E-05	5.E-05	-0.04	0.03	0.05	0.05	-0.30	-0.44	-0.20	-0.30	-0.10	-0.01	0.04	0.03
**cg00469800**	TOR1A	9	132,585	Body	N	5.E-05	9.E-05	-0.04	-0.02	-0.01	0.01	-0.78	-0.89	-0.21	-0.28	-0.33	-0.19	-0.01	0.01
**cg17901584**	DHCR24	1	55,354	TSS1500	rs597021 Body height	5.E-05	1.E-04	-0.31	-0.20	-0.02	-0.05	-0.74	-0.30	-0.30	-0.47	0.07	0.03	-0.01	0.01
**cg04126095**	MASP1	3	186,954	Body	rs3864095 Type 2 diabetes	5.E-05	9.E-05	-0.01	0.00	-0.01	-0.01	-0.79	-0.72	-0.21	-0.35	-0.52	-0.31	0.01	0.00
**cg02612228**	MYOM2	8	1,983		N	5.E-05	3.E-04	-0.01	0.01	0.01	-0.01	-0.76	-0.53	-0.22	-0.40	0.41	0.06	-0.02	0.03
**cg16824319**	LYPD8	1	248,904	TSS1500	N	5.E-05	8.E-05	0.02	0.02	0.00	0.01	-0.64	-0.41	-0.29	-0.49	-0.02	0.03	0.00	-0.05
**cg18000306**	DUSP22	6	289		N	5.E-05	3.E-05	-0.11	-0.04	0.04	-0.05	-0.28	0.05	-0.16	-0.33	0.29	0.25	0.00	0.11
**cg01875529**	ANKRD11	16	89,355	Body	rs57258486 Body height	5.E-05	7.E-05	-0.15	-0.07	0.03	0.02	-0.29	-0.41	-0.17	-0.32	-0.41	-0.14	0.06	0.06
**cg12489353**	EHD2	19	48,231	Body	rs12973221 BMI	5.E-05	7.E-05	0.19	0.06	-0.01	0.05	-0.39	-0.14	-0.22	-0.30	0.18	0.16	0.00	0.02
**cg02593766**	MYCBPAP/EP N3	17	48,609	Body; TSS1500	N	5.E-05	2.E-04	-0.34	-0.23	-0.04	-0.12	-0.27	-0.27	-0.21	-0.40	0.08	0.02	-0.03	0.00
**cg07873325**	KRCC1	2	88,356	TSS1500	N	5.E-05	5.E-04	-0.02	-0.02	0.01	-0.02	-0.40	-0.59	-0.20	-0.30	-1.19	-0.55	0.00	-0.04
**cg10975863**	RAD51B	14	68,831	Body	rs11158743 Body height	6.E-05	3.E-05	0.05	0.05	0.05	0.05	-0.43	-0.32	-0.24	-0.40	-0.07	-0.06	-0.02	-0.03
**cg21324211**	FSD2	15	83,419		N	6.E-05	8.E-05	-0.11	-0.06	0.00	0.04	-0.36	-0.60	-0.23	-0.25	-0.36	0.03	0.03	-0.01
**cg21575634**	STX2	12	131,310	Body	N	6.E-05	2.E-04	0.05	0.05	0.05	0.05	-0.43	-0.32	-0.24	-0.40	-0.12	-0.05	0.00	0.01
**cg20817228**	LOC728855	1	149,615	Body	N	6.E-05	6.E-05	-0.01	0.01	0.01	-0.01	-0.76	-0.53	-0.22	-0.40	-0.04	0.10	0.00	0.00
**cg03112433**	CDK14	7	90,339	TSS200	N	7.E-05	3.E-04	-0.31	-0.20	-0.02	-0.05	-0.74	-0.30	-0.30	-0.47	0.04	0.16	0.01	-0.02
**cg03103888**	TMCO3	13	114,148	5'UTR	N	7.E-05	1.E-04	-0.31	-0.20	-0.02	-0.05	-0.74	-0.30	-0.30	-0.47	-0.09	0.00	0.02	-0.05
**cg16756527**	RCAN1	21	35,891	Body	N	7.E-05	7.E-05	-0.11	-0.06	-0.03	-0.04	-0.66	-0.67	-0.19	-0.26	0.06	-0.06	-0.01	0.02
**cg19755813**	GEMIN4	17	650	Body	N	7.E-05	9.E-05	-0.11	-0.06	-0.03	-0.04	-0.66	-0.67	-0.19	-0.26	0.52	0.20	-0.01	0.02
**cg02386599**	DAAM2	6	39,770	5'UTR	rs1928191 Body height	7.E-05	2.E-04	-0.05	-0.01	0.04	0.04	-0.10	-0.43	-0.12	-0.30	0.00	-0.07	0.02	0.01
**cg14363249**	TMC1	9	75,214	5'UTR	N	7.E-05	1.E-04	-0.01	0.03	-0.02	-0.02	-0.31	-0.42	-0.22	-0.38	-0.03	-0.05	0.00	-0.02
**cg03217337**	CHMP4B	20	32,447		Rs12625495 Body height	7.E-05	1.E-04	0.05	0.00	-0.01	-0.04	-0.71	-0.37	-0.24	-0.34	0.06	0.05	0.00	0.12
**cg10107890**	-	2	44,314		rs4953076 Body height	7.E-05	1.E-04	0.14	0.00	-0.07	-0.07	-0.97	-0.36	-0.19	-0.37	-0.04	-0.04	-0.02	-0.03
**cg07414571**	ZNF322	6	26,690		rs198853 Body height	7.E-05	8.E-05	0.07	0.07	0.05	0.00	-0.35	-0.27	-0.20	-0.27	-0.16	0.23	0.10	-0.10
**cg27457201**	RPTOR	17	78,854	Body	rs11150745 BMI	8.E-05	4.E-04	0.05	0.04	0.01	0.07	-0.71	-0.37	-0.27	-0.46	0.02	-0.07	0.01	-0.01
**cg04580750**	LINC02774	1	244,200		rs12027747 Body height	8.E-05	1.E-04	-0.10	-0.04	0.04	-0.01	-0.61	-0.05	-0.31	-0.47	-0.24	-0.10	0.01	0.00
**cg16678522**	CBFA2T2	20	32,150	Body; TSS1500	rs12625495 Body height	8.E-05	2.E-04	-0.12	-0.06	0.02	0.01	-0.67	-0.53	-0.25	-0.30	-0.13	0.02	0.01	-0.11
**cg25527325**	PLIN5	19	4,524	Body	rs56356382 BMI	8.E-05	4.E-04	-0.03	-0.01	0.02	0.02	-0.46	-0.16	-0.25	-0.47	-0.58	-0.36	-0.05	0.00
**cg08104594**	DRAXIN	1	11,786		rs2336030 BMI	8.E-05	9.E-05	0.15	0.05	-0.09	-0.06	-0.52	0.12	-0.24	-0.35	0.07	0.01	0.00	0.01
**cg24134056**	FLJ12825	12	54,495	Body; TSS1500	Rs12822416 Waist cir cumference	8.E-05	7.E-05	0.02	0.02	0.00	0.01	-0.64	-0.41	-0.29	-0.49	0.07	0.04	0.00	0.00
**cg07658646**	PRR4	12	11,002	TSS200;	N	8.E-05	7.E-05	-0.16	-0.06	0.05	-0.12	-0.46	0.07	-0.40	-0.54	0.06	0.06	0.00	0.06

				Body													
**cg14009440**	TMEM53	1	45,139	Body	Rs781771<br>IL6	8.E-05	2.E-04	-0.16	-0.06	0.05	-0.12	-0.46	0.07	-0.40	-0.54	0.05	0.02	0.00	0.03
**cg04515702**	AKR1B10P1	10	69,510		rs573819939<br>Body weight	8.E-05	1.E-04	-0.02	-0.02	0.01	-0.02	-0.40	-0.59	-0.20	-0.30	0.06	0.11	0.00	-0.04
**cg09010699**	ACAP2	3	195,172		rs149877182<br>Body height	8.E-05	2.E-04	0.14	0.00	-0.07	-0.07	-0.97	-0.36	-0.19	-0.37	0.10	0.13	-0.04	-0.01
**cg13081568**	NKAIN2	6	125,145	3'UTR	rs1871329<br>BMI	9.E-05	2.E-04	0.02	0.02	0.00	0.01	-0.64	-0.41	-0.29	-0.49	0.00	-0.02	-0.01	-0.06
**cg26543333**	NFIC	19	3,398	Body	rs56356382<br>BMI	9.E-05	2.E-04	-0.07	-0.03	0.00	0.02	-0.29	-0.45	-0.16	-0.30	0.20	-0.01	0.00	0.00
**cg20946054**	DRAXIN	1	11,762	5'UTR	rs2336030<br>BMI	9.E-05	7.E-05	-0.06	-0.01	0.06	0.07	-0.27	-0.19	-0.23	-0.38	0.31	0.16	0.01	0.04
**cg13931599**	CUBNP3/<br>ANKRD54P1	10	45,650	TSS200	N	9.E-05	1.E-04	0.05	0.00	-0.01	-0.04	-0.71	-0.37	-0.24	-0.34	0.40	0.17	0.00	-0.09
**cg22941668**	miR-145	5	148,810	TSS200;<br>Body	rs783779<br>Body height	9.E-05	9.E-05	-0.03	-0.01	0.02	0.02	-0.46	-0.16	-0.25	-0.47	0.02	0.08	0.02	-0.20
**cg20814202**	AATK	17	79,129	Body	rs62073017<br>Body height	9.E-05	7.E-05	-0.11	-0.04	0.04	-0.05	-0.28	0.05	-0.16	-0.33	-0.49	-0.33	0.01	-0.01
**cg24465329**	-	2	172,453		rs56228832<br>Lean body mass	9.E-05	6.E-05	-0.04	-0.03	-0.05	-0.05	-0.92	-1.03	-0.22	-0.31	0.10	0.08	-0.01	0.05
**cg11211384**	-	6	142,360		rs6930558<br>Birth weight	9.E-05	1.E-04	0.15	0.05	-0.09	-0.06	-0.52	0.12	-0.24	-0.35	0.06	0.01	0.04	0.03
**cg07603794**	CLASP1	2	122,355	Body	N	1.E-04	8.E-05	0.05	0.05	0.05	0.05	-0.43	-0.32	-0.24	-0.40	0.10	0.05	0.04	-0.01

[†] *P* values adjusted for obesity-related polygenic risk score

[#] Corresponds to estimate of $\beta_M(t)$ in Figure 1. We estimated the average effect of 3 to 5 months of EBF vs. 0 month.

[$] Corresponds to estimate of $\beta_{DIR}(t)$ in Figure 1. We estimated the average effect of 3 to 5 months of EBF vs. 0 month.

[&] Corresponds to estimate of $\beta_{IND}(t)$ in Figure 1. We estimated the average effect of 3 to 5 months of EBF vs. 0 month.

Supplementary Table 2: Top EWAS hits from ALSPAC data in boys selected at the P=1.10^{-4} level.

CpG site	Gene	Chr	Pos.	Group	*GWAS SNP*	*P*	*P*adj†	EBF effect on DNAm (≥3 months vs. 0)#				EBF direct effect on BMI (≥3 months vs. 0)$				EBF effect on BMI mediated by DNAm (≥3 months vs. 0)&			
								1 year	2 years	7 years	15 years	1 year	2 years	7 years	15 years	1 year	2 years	7 years	15 years
**cg26657240**	IL16	15	81,475		rs11325 Body height	3.E-07	5.E-06	-0.02	-0.02	-0.03	-0.03	-1.43	-0.39	-0.01	-0.06	-0.01	0.00	0.05	0.23
**cg08651538**	NREP	5	111,070	Body	rs26543 BMI	4.E-07	7.E-06	-0.06	-0.05	-0.03	0.02	-1.32	-0.42	0.02	-0.07	0.03	0.02	0.00	0.09
**cg26227569**	CPZ/GPR78	4	8,687		N	2.E-06	8.E-07	-0.04	-0.03	-0.02	-0.01	-0.92	-0.62	0.02	-0.10	0.69	0.52	0.01	0.01
**cg10658438**	GP5	3	194,117	3'UTR	N	4.E-06	4.E-05	0.03	0.03	0.11	0.04	-0.55	-0.45	-0.16	-0.31	0.18	0.13	0.02	0.04
**cg22230229**	BAHCC1	17	79,372	TSS1500	rs68007459 Body height	5.E-06	2.E-05	-0.02	-0.02	0.00	0.02	-1.36	-0.43	-0.08	-0.09	0.07	0.02	-0.02	0.05
**cg20559217**	DCPS	11	126,174	TSS200	rs7115672 Body height	6.E-06	3.E-05	-0.01	0.00	0.02	0.00	-1.31	-0.73	0.00	-0.09	0.69	0.45	0.05	-0.01
**cg18436737**	CTBP1	4	1,234	Body	rs72501962 Type 2 diabetes	7.E-06	1.E-04	0.19	0.12	-0.05	0.01	-1.51	-0.71	0.08	-0.17	-0.04	0.00	0.02	0.09
**cg27149179**	ME3/ PRSS23	11	86,452		rs4944571 obesity	8.E-06	2.E-05	-0.35	-0.26	-0.04	-0.04	-0.79	-0.70	0.09	-0.34	0.83	0.23	-0.01	0.05
**cg01972418**	PAX9	14	37,125	TSS1500	rs10131337 Body height	9.E-06	2.E-05	0.01	-0.01	-0.06	-0.03	-1.01	-0.66	0.05	-0.14	0.16	0.05	-0.02	0.09
**cg15185570**	BARX2	11	129,488		N	1.E-05	5.E-06	-0.02	-0.02	-0.01	0.01	-0.88	-0.67	0.01	-0.20	-0.08	-0.02	0.03	-0.01
**cg05145608**	FOXK1	7	4,758	Body	N	1.E-05	8.E-05	-0.06	-0.04	-0.02	-0.08	-0.73	-0.65	0.04	-0.09	-0.04	-0.08	-0.01	-0.03
**cg09552166**	MSL2	3	135,913	TSS200; Body	rs372030255 Body height	1.E-05	3.E-05	0.08	0.08	0.08	0.01	-1.01	-0.53	0.02	-0.05	-0.77	-0.05	-0.01	0.05
**cg08079596**	ATOH8	2	86,000	Body	N	1.E-05	1.E-04	-0.01	-0.03	-0.04	-0.02	-1.31	-0.78	0.14	0.01	-0.01	0.00	0.00	0.03
**cg02640147**	KCNIP1	5	170,025	Body	N	1.E-05	3.E-04	0.02	0.01	-0.05	-0.01	-1.15	-0.67	0.05	-0.02	-0.04	-0.02	0.00	0.07
**cg09188763**	LMO1	11	8,286	TSS1500	rs110419 Body height	1.E-05	5.E-05	0.01	-0.02	-0.03	-0.06	-1.21	-0.75	0.04	0.02	0.45	0.24	-0.01	0.04
**cg07143733**	miR155HG-miR155	21	26,939	Body	N	2.E-05	4.E-05	-0.20	-0.19	-0.01	-0.08	-1.96	-0.38	-0.11	-0.15	-0.44	-0.18	-0.05	0.05
**cg22792674**	VPS72	1	151,164	TSS1500	rs4512652 BMI	2.E-05	24.E-05	0.00	0.00	0.00	0.00	-1.39	-0.82	0.03	-0.10	0.01	-0.02	-0.01	-0.10
**cg17765387**	FBXW8	12	117,355	Body	rs12823620 Body height	2.E-05	2.E-05	-0.26	-0.19	-0.03	-0.07	-0.34	-0.17	0.02	-0.25	-0.15	0.11	0.00	-0.07
**cg19865725**	RTN4	2	55,321		rs7582359 BMI	2.E-05	1.E-04	-0.07	-0.04	0.04	-0.04	-0.95	-0.68	0.01	-0.15	-0.14	-0.04	-0.02	-0.17
**cg03389890**	ABCD1P4	22	16,868		N	2.E-05	4.E-05	-0.10	-0.04	0.04	0.03	-2.19	-0.99	0.06	-0.03	0.11	0.23	0.02	-0.09
**cg13915886**	KCNT1	9	138,637	Body	N	2.E-05	1.E-04	-0.03	-0.03	-0.01	-0.04	-1.27	0.09	-0.13	-0.08	0.40	0.32	-0.03	0.02
**cg17945323**	SLC22A8	11	62,767	Body	rs11231299 Adipose tissue	2.E-05	1.E-04	0.03	0.02	-0.04	0.01	-2.35	-0.40	-0.10	-0.19	0.33	0.13	0.00	-0.02
**cg23864514**	TOMM7/ IL6	7	22,883		rs112330055 Body height	2.E-05	6.E-05	-0.02	-0.03	-0.02	-0.02	-1.04	-0.38	-0.07	-0.10	0.11	0.01	-0.06	0.06
**cg09187933**	AC079949.3	12	127,631		N	2.E-05	5.E-05	0.02	0.04	0.10	-0.03	-0.81	-0.15	-0.03	-0.02	-0.03	-0.07	-0.07	0.01

**cg21692620**	CCR10/ CNTNAP1	17	42,683	Body	rs4792953 Body height	2.E-05	2.E-04	-0.03	0.00	0.01	0.06	-1.11	-0.78	0.05	0.03	4.71	1.01	0.00	0.10
**cg23847109**	COL9A2	1	40,783	TSS200	rs1846158 Body height	2.E-05	4.E-04	0.25	0.14	-0.05	-0.05	-2.25	-0.72	-0.24	-0.33	-0.88	-0.46	0.00	0.02
**cg21550785**	ALK	2	29,439	Body	rs1406230 Type 2 diabetes	2.E-05	3.E-04	0.41	0.30	-0.01	-0.02	-1.12	-0.40	0.00	-0.09	-0.01	0.00	-0.01	-0.03
**cg03253268**	CRYAA2	21	44,593	3'UTR	Rs574083674 Waist cir cumference	2.E-05	1.E-05	0.05	0.02	-0.05	0.03	-1.22	-0.47	0.01	-0.02	-0.83	-0.54	-0.01	-0.26
**cg17431280**	ARHGAP35	19	47,507	3'UTR	N	2.E-05	2.E-04	-0.12	-0.10	-0.08	0.06	-0.92	-0.36	0.04	-0.18	-0.02	-0.01	0.00	0.01
**cg17868723**	FGF14	13	102,926	Body	rs2607653 Type 2 diabetes	2.E-05	7.E-05	-0.03	-0.03	-0.01	-0.07	-0.86	-0.29	-0.10	-0.28	-0.46	-0.19	-0.01	0.22
**cg04781638**	CPEB1	15	83,241	TSS1500; Body	rs150684763 Body height	2.E-05	1.E-06	0.00	-0.01	-0.01	-0.01	-1.21	-0.36	-0.04	-0.01	-0.30	-0.11	0.03	0.17
**cg22897141**	MEN1	11	64,810	Body	rs3782089 Body height	3.E-05	2.E-04	-0.49	-0.35	0.05	0.00	-0.58	-0.11	-0.13	-0.24	-0.11	-0.08	0.00	0.01
**cg04709822**	RPS14	5	149,824	Body	Rs113681861 Waist-hip ratio	3.E-05	1.E-04	-0.38	-0.25	0.04	0.00	-0.07	-0.32	-0.03	-0.12	-1.42	-0.40	0.01	0.00
**cg25225655**	MSI2	17	55,469	Body	rs703862 Body height	3.E-05	4.E-04	0.14	0.09	-0.02	-0.03	-1.04	-0.40	-0.02	-0.22	-0.35	-0.11	-0.04	0.07
**cg25494075**	NCAM1	11	112,752		rs1816537 BMI	3.E-05	2.E-04	-0.31	-0.22	-0.02	0.00	-1.06	-0.51	0.10	-0.05	-0.09	-0.23	-0.04	0.16
**cg12765567**	miR-7159/ miR-1275	6	33,919		N	3.E-05	6.E-05	-0.01	-0.01	-0.03	0.01	-1.61	-0.47	-0.05	-0.13	-0.57	-0.20	-0.05	0.01
**cg27122431**	CDH18	5	19,989	TSS1500	rs80240198 Type 2 diabetes	3.E-05	2.E-04	-0.09	-0.07	0.06	0.13	-1.46	-0.38	-0.18	-0.33	0.11	0.09	-0.03	0.03
**cg15689991**	RASSF3	12	65,019	Body	N	3.E-05	3.E-04	0.03	0.02	-0.03	-0.05	-1.64	-0.73	-0.03	0.06	-0.01	0.02	-0.02	-0.01
**cg13585930**	NPFFR1	10	72,027	TSS1500	rs4747011 Body height	4.E-05	5.E-05	-0.06	0.00	0.03	0.00	-0.92	-0.58	0.04	-0.23	-0.41	-0.16	0.02	0.07
**cg19822851**	NR2C2AP	19	19,315	TSS1500	rs11670016 Waist-hip ratio	4.E-05	2.E-04	-0.17	-0.11	0.03	-0.04	-0.45	-0.89	-0.04	-0.28	0.09	0.02	-0.03	0.00
**cg24924449**	IPO5/RAP2A	13	98,508		N	4.E-05	4.E-05	-0.10	-0.09	-0.04	0.01	-0.81	-0.46	-0.05	-0.09	-0.96	-0.42	-0.01	-0.02
**cg17434643**	MIR124-3	20	61,805		N	4.E-05	1.E-04	0.09	0.05	-0.01	0.10	-1.27	-0.61	0.02	-0.24	0.05	0.01	0.03	0.10
**cg19509778**	FOXI2	10	129,537	3'UTR	N	4.E-05	9.E-05	0.09	0.08	0.05	0.06	-1.30	-0.66	0.05	-0.04	-5.63	-2.77	-0.03	0.08
**cg05694290**	HIP1R	12	123,335	Body	rs11060344 Waist-hip ratio	4.E-05	4.E-05	-0.01	-0.02	-0.08	0.06	-2.07	-0.38	-0.22	-0.33	0.32	0.19	0.00	0.25
**cg26938334**	SERPINA1	14	94,850	5'UTR	rs28929474 Body height	4.E-05	6.E-05	-0.03	-0.03	0.07	0.21	-1.42	-0.39	-0.01	0.15	0.08	0.03	-0.01	0.08
**cg22284030**	ARFGAP2	11	47,186	3'UTR	rs10501320 Insulin measurement	4.E-05	6.E-05	0.29	0.22	-0.04	-0.06	-1.63	-1.01	0.04	-0.06	-0.02	-0.03	-0.02	0.07
**cg13748564**	COL27A1	9	116,917	TSS1500	rs10817582 Body height	4.E-05	4.E-04	0.20	0.10	-0.05	-0.02	-1.59	-0.28	-0.09	-0.13	0.03	0.02	-0.01	-0.05
**ch.15.1141100F**	PKM	15	72,509	Body	Rs181706692 Hip circum	4.E-05	7.E-05	-0.05	-0.04	0.01	0.03	-0.76	-0.63	0.10	-0.01	0.08	0.08	-0.05	0.12

					ference												
**cg25477843**	PARP10	8	145,061	Body	rs11783655 Body height	4.E-05	2.E-04	-0.13	-0.10	-0.01	0.05	-0.74	-0.60	-0.01	0.03	0.02	0.01	0.00	-0.02
**cg17395800**	CACNA1F	X	49,088	Body	N	4.E-05	8.E-05	-0.03	-0.04	-0.06	0.05	-0.92	0.03	-0.06	-0.12	-0.94	-0.66	0.00	0.10
**cg15797834**	STAP2	19	4,339	TSS200	N	4.E-05	1.E-04	0.18	0.13	0.02	0.10	-1.71	-0.77	0.05	0.00	-0.01	0.00	-0.02	-0.01
**cg08443605**	RARB	3	24,872		N	5.E-05	2.E-04	0.07	0.04	-0.01	-0.01	-1.49	-0.55	-0.08	-0.09	2.46	0.71	0.01	0.00
**cg05475524**	ACTN3	11	66,327	Body	rs679228 Body height	5.E-05	4.E-04	0.04	0.02	0.03	-0.03	-0.47	-0.34	-0.16	-0.21	0.60	0.18	0.00	0.04
**cg22464447**	IQGAP1/CRTC3	15	91,054		rs11609 Body height	5.E-05	4.E-04	0.00	0.00	-0.02	-0.03	-1.16	-0.55	-0.02	-0.20	0.18	0.12	0.00	-0.13
**cg03569876**	CSNK1A1/ miR143- miR145	5	148,908	Body	rs368510 Body height	5.E-05	2.E-04	-0.32	-0.22	0.04	-0.03	0.41	0.17	0.04	-0.17	-0.02	0.06	0.00	-0.04
**cg19842091**	RASGEF1C	5	179,567	5'UTR	rs4290977 Body height	5.E-05	5.E-05	0.03	0.03	0.07	0.02	-1.13	-0.59	-0.11	-0.12	0.35	0.05	0.00	0.01
**cg06483432**	KIT	4	55,523	TSS1500	rs11727273 BMI	5.E-05	2.E-04	-0.33	-0.24	-0.01	0.09	-0.79	-0.40	-0.03	-0.16	-0.17	0.00	-0.03	0.00
**cg25507226**	DNAJB6	7	157,191	Body	rs1182436 Type 2 diabetes	5.E-05	3.E-04	-0.10	-0.07	-0.01	0.02	-1.09	-0.64	-0.01	-0.01	-0.45	-0.22	0.00	0.16
**cg17141902**	NINJ1	9	95,897	TSS1500	rs13810828 Body height	6.E-05	2.E-05	0.03	0.02	-0.02	-0.05	-2.50	-0.60	-0.27	-0.30	-0.09	-0.04	0.01	-0.02
**cg10222361**	USP31	16	23,071		N	6.E-05	5.E-04	-0.10	-0.06	0.03	-0.06	-1.08	-0.72	0.01	0.03	-0.04	-0.02	-0.05	0.03
**cg26479868**	TMTC1	12	29,916	Body	rs188945888 Hip circum ference	6.E-05	2.E-04	-0.12	-0.10	-0.05	-0.06	-0.78	-1.11	0.04	-0.15	-0.02	0.01	-0.05	-0.20
**cg05738687**	RFLNB	17	290	3'UTR	rs77612020 Waist circum ference	6.E-05	9.E-05	-0.07	-0.05	0.02	0.17	-1.73	-0.63	-0.08	-0.18	0.00	0.02	0.00	0.03
**cg25591451**	TRIM4	7	99,518	TSS1500	rs11973801 BMI	6.E-05	1.E-04	0.05	0.06	0.18	-0.05	-1.16	-0.78	-0.06	-0.17	-0.14	0.02	0.00	0.05
**cg25104430**	ALKBH1	14	78,128		N	6.E-05	1.E-04	-0.15	-0.12	-0.04	-0.09	-0.92	-0.51	0.14	0.02	-0.02	-0.02	0.00	0.05
**cg05895666**	SLC25A43	X	118,533	TSS1500	rs3848873 Body height	6.E-05	2.E-04	-0.07	-0.04	0.04	0.01	-1.01	-0.63	-0.08	-0.06	0.27	0.45	-0.04	0.00
**cg26507353**	MAP3K8/LYZL2	10	30,843		N	7.E-05	4.E-04	0.04	0.03	0.02	0.05	-1.46	-0.08	-0.06	-0.12	-0.33	-0.18	0.03	0.16
**cg03048432**	NIN	14	51,291	5'UTR	N	7.E-05	2.E-04	0.30	0.21	-0.01	0.00	-0.80	-0.58	0.03	-0.06	-0.95	-0.31	0.02	0.12
**cg12394014**	MORF4L1	15	79,172	Body	N	7.E-05	7.E-04	0.04	0.02	0.00	-0.02	-0.73	-0.67	0.10	0.03	0.13	0.10	-0.02	-0.02
**cg13478737**	FIBCD1	9	133,807	Body	rs3739514 BMI	8.E-05	2.E-04	0.88	0.64	0.02	-0.13	-0.56	0.11	-0.01	-0.33	0.05	0.02	-0.04	0.01
**cg07798347**	RAP1GAP	1	21,918		Rs55722102 Body height	8.E-05	6.E-05	0.22	0.14	-0.02	0.10	-1.30	-0.51	-0.06	-0.29	-1.20	-0.36	0.00	0.05
**cg17929273**	LGALS2	22	37,968	Body	N	8.E-05	3.E-04	-0.12	-0.09	0.03	-0.05	-1.65	-0.53	-0.12	-0.18	0.10	0.00	0.02	0.17
**cg09469566**	SOX1	13	112,717		N	8.E-05	7.E-04	0.02	0.01	-0.02	-0.01	-1.13	-0.85	0.05	-0.03	-0.02	-0.01	0.00	-0.07
**cg15661389**	GAS7	17	10,019	Body	rs2320549 Body height	8.E-05	2.E-04	-0.23	-0.17	0.02	-0.02	0.03	-0.46	-0.12	-0.38	-0.16	-0.06	-0.02	0.12
**cg01381374**	GNGT1	7	93,474		rs2528531 BMI	8.E-05	1.E-04	-0.05	-0.03	0.04	-0.07	-1.18	-0.47	-0.14	-0.16	1.01	0.30	0.00	0.01
**cg10614809**	COBL	7	51,539		rs11765748 BMI	8.E-05	3.E-04	-0.17	-0.16	-0.02	-0.03	-1.08	-0.70	0.08	0.10	0.15	0.05	0.00	0.00
**cg07951931**	GALNT9	12	132,886	Body	rs11247009	8.E-05	8.E-05	-0.40	-0.30	-0.02	-0.01	-1.42	-0.99	0.06	-0.05	-0.29	-0.25	0.00	0.02

					BMI												
**cg01919939**	DPH6	15	35,990		rs7181610 BMI	8.E-05	2.E-04	-0.27	-0.19	-0.01	-0.08	-0.63	-1.03	0.01	-0.09	3.06	1.29	0.01	0.00
**cg13882795**	NRARP	9	140,195	1stExon; 3'UTR	N	8.E-05	9.E-05	0.18	0.14	0.11	0.09	-0.68	-0.59	0.07	-0.02	-0.01	0.07	-0.01	0.07
**cg15661753**	TUBA1C	12	49,627		N	8.E-05	4.E-04	-0.17	-0.13	0.00	-0.02	-1.02	-0.31	-0.07	-0.09	-1.38	-0.54	0.01	0.00
**cg20276677**	NPSR1	7	34,698	TSS200	rs73310815 Obesity	9.E-05	2.E-04	-0.29	-0.18	0.05	0.02	-0.61	-0.81	-0.02	-0.19	-0.29	-0.16	0.01	0.01
**cg18335290**	LIMS2	2	128,439	1stExon; 5'UTR	rs73954691 Type 2 diabetes	9.E-05	4.E-04	0.05	0.03	0.03	-0.04	-0.87	-0.12	-0.02	-0.13	0.14	0.05	0.00	0.05
**cg17566334**	PACRG	6	163,515	Body	N	9.E-05	9.E-05	0.00	0.00	-0.01	0.03	-1.36	-0.54	0.09	-0.09	0.00	-0.01	-0.03	-0.14
**cg11704631**	RUNX1	21	36,396	Body	Rs4507181 Waist-hip ratio	9.E-05	4.E-04	-0.19	-0.14	0.07	0.03	-1.03	-0.63	0.00	-0.13	-0.13	0.00	-0.05	0.06
**cg07341022**	FOXD4L1	2	114,269		N	9.E-05	4.E-04	-0.01	-0.01	-0.05	-0.13	-1.01	-0.74	0.06	-0.02	-0.34	-0.23	0.01	-0.07
**cg02129885**	ID1/ miR3193	20	30,218		N	1.E-04	2.E-04	0.23	0.15	-0.06	0.06	-2.13	-0.90	0.00	-0.15	-0.13	-0.10	-0.01	0.03
**cg02324580**	SRCAP	16	30,751	Body	rs4402589 BMI	1.E-04	2.E-03	0.08	0.07	0.09	-0.12	-1.08	-0.50	-0.05	-0.13	-0.17	-0.09	0.00	0.12
**cg01260615**	ZNF184	6	27,485		N	1.E-04	2.E-04	-0.13	-0.11	-0.02	-0.05	-1.25	-0.57	0.07	-0.02	0.18	0.11	0.01	-0.09
**cg01877937**	L3MBTL	20	42,143	TSS1500	N	1.E-04	3.E-04	0.18	0.11	0.02	-0.02	-1.19	-0.64	0.02	0.00	0.11	-0.03	0.00	0.01

[†] *P* values adjusted for obesity-related polygenic risk score

[#] Corresponds to estimate of $\beta_{M}(t)$ in Figure 1. We estimated the average effect of 3 to 5 months of EBF vs. 0 month.

[$] Corresponds to estimate of $\beta_{DIR}(t)$ in Figure 1. We estimated the average effect of 3 to 5 months of EBF vs. 0 month.

[&] Corresponds to estimate of $\beta_{IND}(t)$ in Figure 1. We estimated the average effect of 3 to 5 months of EBF vs. 0 month.

Supplementary Table 3. CpG sites selected in multi-mediator analyses based on forward selection. The P-value to enter the model is 10^{-4} and to stay in the model 10^{-3}.

Girls			Boys		
**CpG site**	Gene	P-value[#]	CpG site	Gene	P-value[#]
**cg06471491**	miR-21	$<10^{-16}$	cg26657240	IL16	$<10^{-16}$
**cg16645539**	SNAPC3	$5.6x10^{-16}$	cg08651538	NREP	$6.1x10^{-10}$
**cg04126095**	MASP1	$2.5x10^{-14}$	cg20559217	DCPS	$1.3x10^{-9}$
**cg10082165**	CTSG	$6.6x10^{-10}$	cg07951931	GALNT9	$1.7x10^{-9}$
**cg26872564**	ZNF185	$1.2x10^{-8}$	cg26227569	CPZ/GPR78	$9.4x10^{-8}$
**cg00524694**	PROP1	$2.3x10^{-7}$	cg01972418	PAX9	$1.7x10^{-7}$
**cg26992566**	EMC8	$3.1x10^{-7}$	cg07143733	miR155HG-miR155	$9.0x10^{-7}$
**cg17401282**	GABRG2	$5.0x10^{-7}$	cg19509778	FOXI2	$1.4x10^{-6}$
**cg07603794**	CLASP1	$1.6x10^{-4}$	cg22230229	BAHCC1	$8.0x10^{-6}$
**cg17901584**	DHCR24	$2.7x10^{-4}$	cg23847109	COL9A2	$3.2x10^{-5}$
**cg11746359**	MAML3	$6.4x10^{-4}$	cg19822851	NR2C2AP	$1.5x10^{-4}$
**cg07012178**	PRKAG2	$9.2x10^{-4}$			
**cg03103888**	TMCO3	$9.7x10^{-4}$			

[#]P-value in the final model.

Supplementary Table 4. Functional annotation clustering with DAVID, significant terms at the 5% level

Category	Term	Number of Genes	*P-value*
**Girls**			
KEGG_PATHWAY	AMPK signaling pathway	4	0.034
KEGG_PATHWAY	Insulin signaling pathway	4	0.046
KEGG_PATHWAY	Endocytosis	5	0.050
GOTERM	ATP binding	14	0.019
GOTERM	Multicellular organism development	7	0.049
**Boys**			
KEGG_PATHWAY	Pathways in cancer	7	0.025
GOTERM	Negative regulation of transcription, DNA-templated	10	$1E10^{-4}$
GOTERM	Positive regulation of tyrosine phosphorylation of Stat3 protein	3	0.020
GOTERM	Negative regulation of transcription from RNA polymerase II promoter	9	0.031
GOTERM	Regulation of cell shape	4	0.035
GOTERM	Hemopoiesis	3	0.036

Supplementary Table 5: Top miRNAs associated with child longitudinal BMI from ALSPAC data in boys and girls selected at the P=1.10^{-4} level.

CpG site	miRNA	Chr	Pos.	Breast Milk[†]	*P*	EBF effect on DNAm (≥3 months vs. 0)[#]				EBF direct effect on BMI (≥3 months vs. 0)[$]				EBF effect on BMI mediated by DNAm (≥3 months vs. 0)[&]			
			(kb)			1 year	2 years	7 years	15 years	1 year	2 years	7 years	15 years	1 year	2 years	7 years	15 years
**Girls**																	
**cg06471491**	miR-21	17	57,918	**Yes**	<E-16	0.02	-0.08	-0.07	-0.07	-1.13	-0.51	-0.17	-0.37	0.36	0.11	0.00	0.01
**cg05241732**	1Kb from miR-208a	14	23,859	**No**	3.E-06	-0.20	-0.12	0.03	0.00	-0.28	-0.22	-0.26	-0.39	-0.04	-0.02	-0.01	0.01
**cg06527213**	1Kb from miR-1276	15	86,315	**Yes**	4.E-05	-0.04	-0.03	-0.05	-0.05	-0.92	-1.03	-0.22	-0.31	-0.13	-0.03	-0.02	-0.09
**cg22941668**	miR-145	5	148,810	**Yes**	9.E-05	-0.03	-0.01	0.02	0.02	-0.46	-0.16	-0.25	-0.47	0.02	0.08	0.02	-0.20
**Boys**																	
**cg07143733**	miR-155HG	21	26,939	**Yes**	2.E-05	-0.20	-0.19	-0.01	-0.08	-1.96	-0.38	-0.11	-0.15	-0.44	-0.18	-0.05	0.05
**cg12765567**	2Kb from miR-7159	6	33,919	**No**	3.E-05	-0.01	-0.01	-0.03	0.01	-1.61	-0.47	-0.05	-0.13	-0.57	-0.20	-0.05	0.01
**cg17434643**	4Kb from miR-124-3	20	61,805	**Yes**	4.E-05	0.09	0.05	-0.01	0.10	-1.27	-0.61	0.02	-0.24	0.05	0.01	0.03	0.10
**cg22941668**	miR-145	5	148,810	**Yes**	5.E-05	-0.32	-0.22	0.04	-0.03	0.41	0.17	0.04	-0.17	-0.02	0.06	0.00	-0.04

[†]Expressed in breast milk according to database liqDB

[#]Corresponds to estimate of $\beta_M(t)$ in Figure 1. We estimated the average effect of 3 to 5 months of EBF vs. 0 month.

[$]Corresponds to estimate of $\beta_{DIR}(t)$ in Figure 1. We estimated the average effect of 3 to 5 months of EBF vs. 0 month.

[&]Corresponds to estimate of $\beta_{IND}(t)$ in Figure 1. We estimated the average effect of 3 to 5 months of EBF vs. 0 month.

Supplementary Table 6: mQTL analaysis for the top EWAS hits based on the mQTLdb database[37]

CpG site	Gene	Chr	Pos. (kb)	mQTL	*SNP Chr*	*SNP position*	*Cis/Trans*	*Epoch*
**Girls**								
**cg06471491**	miR-21	17	57,918	rs4742392	9	789	Trans	Childhood
**cg16645539**	SNAPC3	9	15,423	-	-	-	-	-
**cg07408552**	ATP6V0A1	17	40,612	Multiple	6	29,918-31,457	Trans	Birth/Childhood/Adolescence
**cg08224066**	DHX15/ PPARGC1A	4	24,578	-	-	-	-	-
**cg14822546**	LINC00398/ ALOX5AP	13	31,381	-	-	-	-	-
**cg14776321**	FAM238C	10	27,220	-	-	-	-	-
**ch.8.18261148F**	NATP/NAT2	8	18,217	-	-	-	-	-
**cg00313685**	CUX1	7	101,469	Multiple	7	101,460-101,498	Cis	Childhood/Adolescence
**cg17476951**	TRAPPC9	8	141,054	Multiple	8	141,047	Cis	Birth
**-**	-	-	-	Multiple	11	38,646-38,984	Trans	Adolescence
**-**	-	-	-	Multiple	1	47,129-47,178	Trans	Adolescence
**-**	-	-	-	Multiple	8	141046- 141047	Cis	Birth
**cg26398656**	OSBPL1A	18	21,852	Multiple	16	79563- 79568	Trans	Childhood
**-**	-	-	-	rs34918972	18	21896	Cis	Adolescence
**-**	-	-	-	Multiple	8	76716- 77199	Trans	Birth
**-**	-	-	-	Multiple	15	97556- 79569	Trans	Birth
**cg26872564**	ZNF185	X	152,087	Multiple	X	152059-152105	Cis	Birth/Childhood/Adolescence
**cg18176842**	FAM84A	2	14,776	Multiple	9	131985- 131982	Trans	Birth
**cg04354689**	PDPK1	16	2,660	-	-	-	-	-
**Boys**								
**cg26657240**	IL16	15	81,475	Multiple	15	81465- 81667	Cis	Childhood/Adolescence
**cg08651538**	NREP	5	111,070	-	-	-	-	-

Supplementary Table 7: Distribution of the main epidemiological variables in Gen3G

	Boys	Girls
	N=104	N=89
**Duration of exclusive breastfeeding (EXBF) (in months)**		
0	22 (21.2%)	22 (24.7%)
1	15 (14.4%)	3 (3.4%)
2	11 (10.6%)	7 (7.9%)
3	6 (5.8%)	6 (6.7%)
4	15 (14.4%)	12 (13.5%)
≥5	35 (33.7%)	39 (43.8%)
**Mother's Education**		
Lower than college	24 (24.1%)	25 (28.1%)
College or higher	80 (76.9%)	64 (71.9%)
**Mother's ever smoking status**		
No	97 (93.3%)	83 (93.3%)
Yes	7 (6.7%)	6 (6.7%)
**Child BMI at birth (in kg/m^2)**		
Mean (±SD)	13.0 (±1.1)	13.1 (±1.1)
**Mother's BMI (1st semester of pregnancy) (in kg/m^2)**		
Mean (±SD)	26.1 (±6.6)	24.9 (±4.9)
**Gestational age (in weeks)**		
Mean (±SD)	39.6 (1.1)	39.5 (1.1)
**Ethnic group**		
Caucasians	101 (97.1%)	85 (95.5%)
Non-Caucasians	3 (2.9%)	4 (4.5%)

Supplementary Table 8: Replication of top EWAS hits associated with child longitudinal BMI in Gen3G data at 5 years of age (at the P=2.9x10^{-4} level)

CpG site	Gene	Chr	Pos. (kb)	*P-value*	EBF effect on DNAm (≥3 months vs. 0)	EBF direct effect on BMI (≥3 months vs. 0)	EBF effect on BMI mediated by DNAm (≥3 months vs. 0)
**Girls**							
**cg22879191**	CUX1	7	101,469	<8E-05	-0.033	-0.57	0.03
**cg19769982**	miR-155HG	21	26,934	1.7x10^{-4}	-0.003	0.24	-0.001

Extended data figures

Extended data Fig.1. Mediation model for the association between exclusive breastfeeding (EBF) and child BMI from birth to 18 years of age. EXP(t) represents the estimate of EBF duration on DNAm at age *t*. NIE(t) represents the indirect effect of EBF duration on child BMI at age *t* mediated by DNAm (see Methods section). NDE(t) represents the direct effect of EBF on child BMI at age *t* not mediated by DNAm. TOT(t) represents the total effect of EBF on child BMI at age *t* (i.e., direct + indirect effects). The top CpG sites were selected based on the M(t)*E(t) interaction effect on y(t), that is the overall interaction effect on child BMI from birth to 18 years.

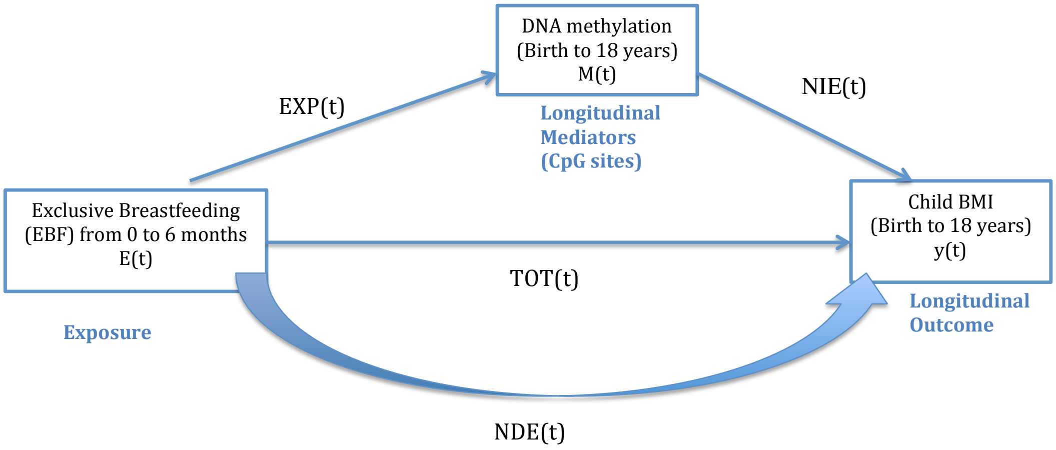

Extended data Fig.2. Genomic landscape of the EWAS-significant CpG site cg06471491 in *miR-21* discovered in ALSPAC.

The *H3K4Me1*, *H3K27Ac* and *H3K4Me3* tracks show histone modifications, suggestive of regulatory activities. The DNase hypersensitivity track shows regions hypersensitive to cutting by the DNase enzyme. Regulatory regions are generally DNase sensitive. Transcription factor (TF) binding track displays regions of TF (proteins responsible for modulating gene transcription) binding. The DNA regulatory elements track displays the chromatin states, where different colors represent different annotations. Bright red predicted promoter region including TSS; The CpG tracks display the methylation status of specific CpGs in blood cell line and embryonic stem cell line, respectively, where the methylation status is color coded. Orange is methylated; purple is partially methylated; blue is unmethylated.

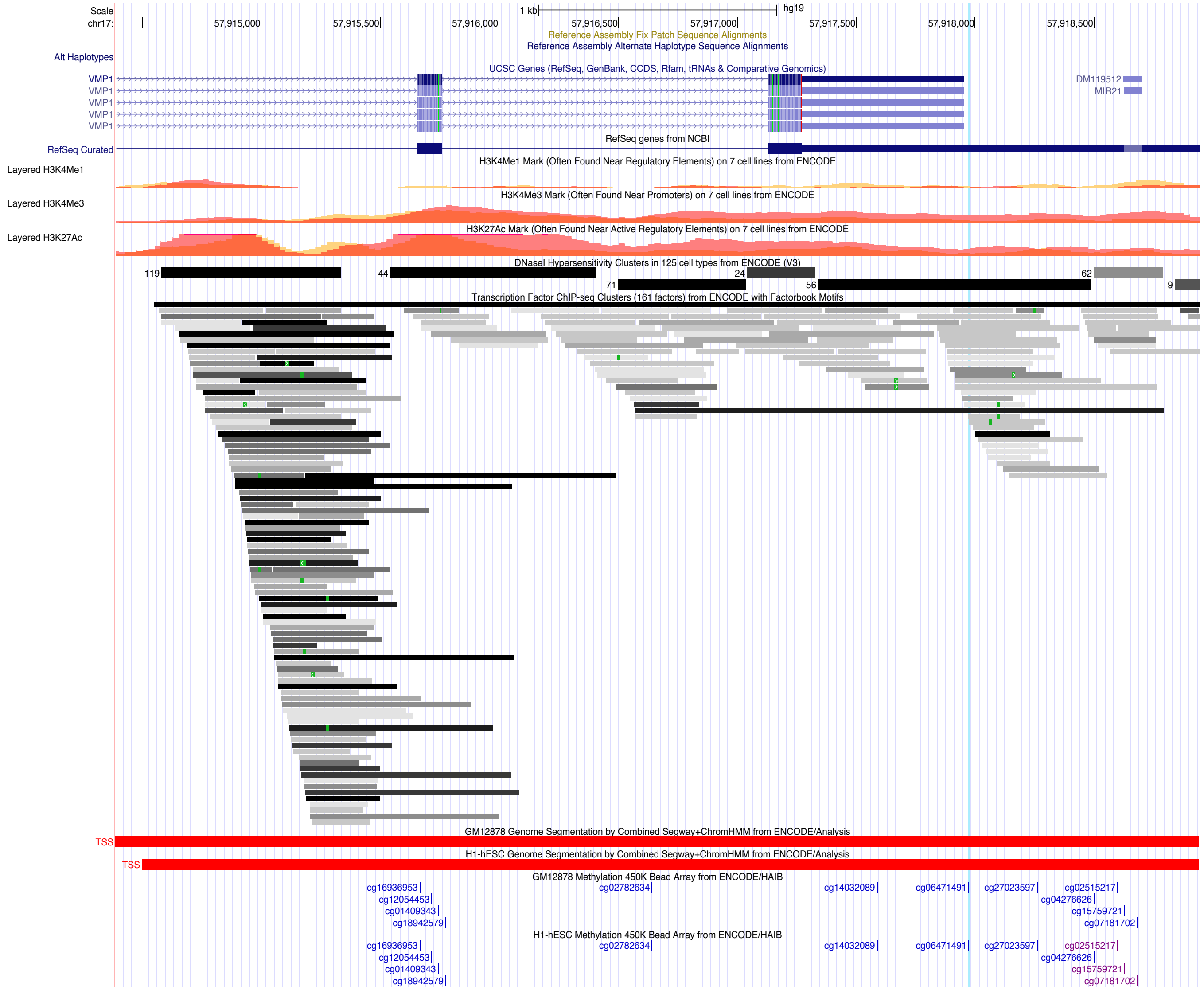

Extended data Fig.3. Genomic landscape of the EWAS-significant CpG site cg07143733 discovered in ALSPAC and cg19769982 found in GEN3G in *miR-155HG.*

The *H3K4Me1*, *H3K27Ac* and *H3K4Me3* tracks show histone modifications, suggestive of regulatory activities. The DNase hypersensitivity track shows regions hypersensitive to cutting by the DNase enzyme. Regulatory regions are generally DNase sensitive. Transcription factor (TF) binding track displays regions of TF (proteins responsible for modulating gene transcription) binding. The DNA regulatory elements track displays the chromatin states, where different colors represent different annotations. Bright red predicted promoter region including TSS; Orange predicted enhancer; blue predicted CTCF (CCCTC-binding factor) enriched element; gray predicted repressed or low activity region; Green predicted a transcribed region. The two CpG tracks display the methylation status of specific CpG in blood cell line and embryonic stem cell line, respectively, where the methylation status is color coded. Orange is methylated; purple is partially methylated; blue is unmethylated.

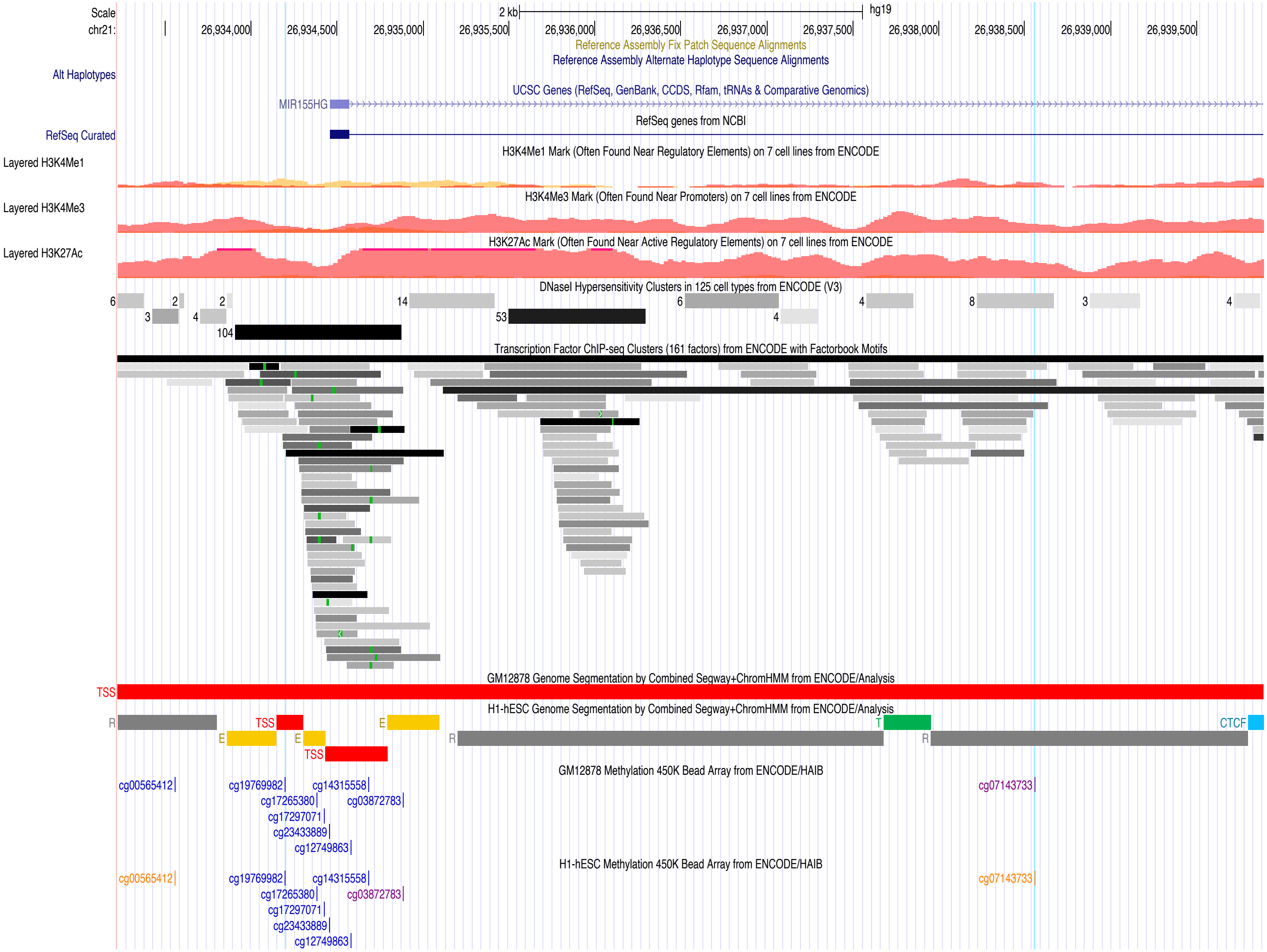

Extended data Fig.4. Gene-gene and gene-miRNA networks built from CpG sites mediating the association between EBF and child BMI. (The red nodes correspond to genes and blue nodes to miRNAs discovered in our analysis. The grey nodes correspond to other known miRNAs not discovered in our analysis).

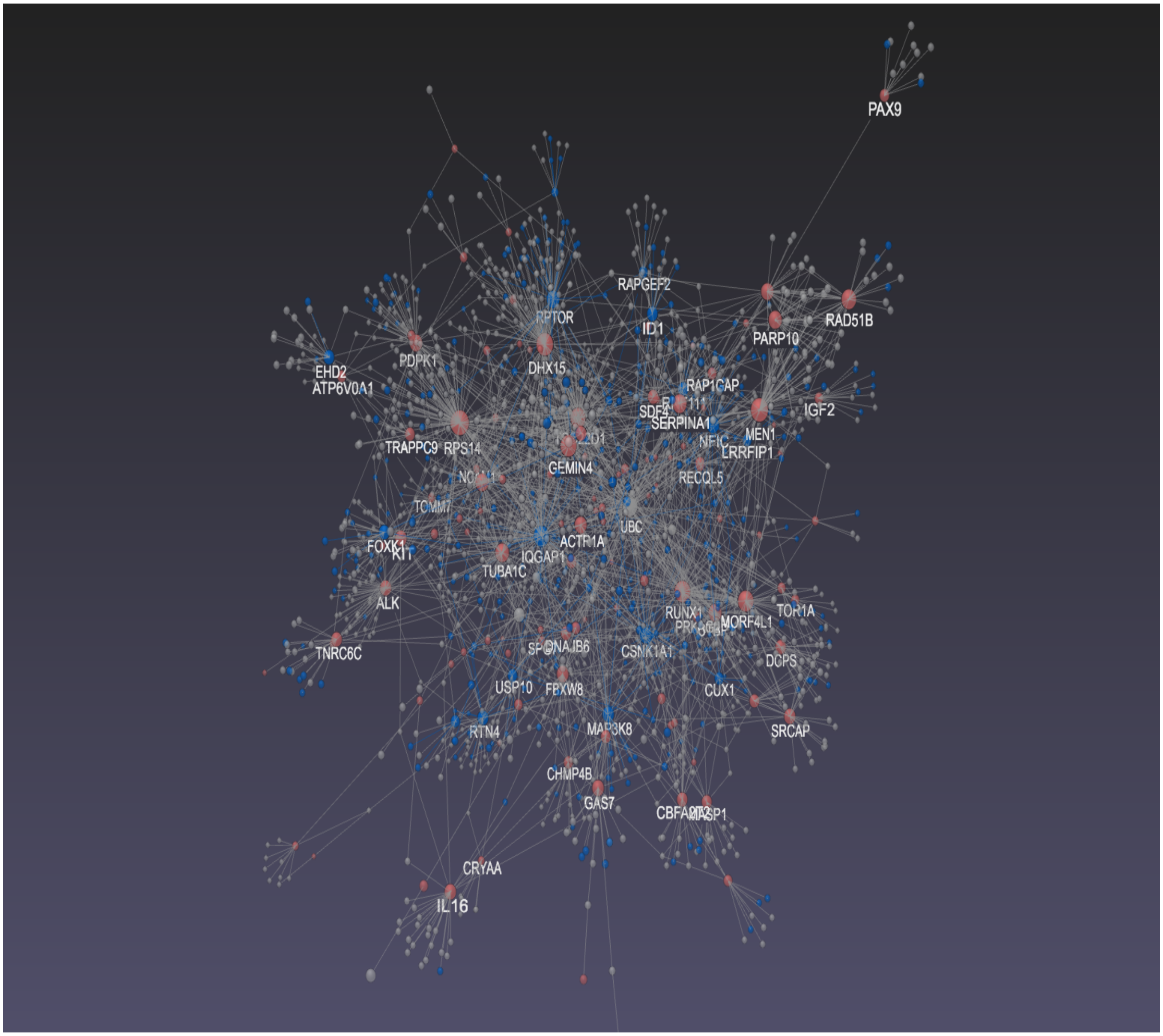

Extended data Fig.5. Change in BMI mediated by DNA methylation from the combined effect of multiple CpG sites where DNA methylation is induced by different durations (3,4 and 5 months vs. no breastfeeding) of exclusive breastfeeding (EBF) in ALSPAC girls and boys, at different ages from birth to 17 years, and after adjustment for BMI-related polygenic risk score. The CpG sites in the final multi-mediator model were selected by forward selection (see Methods). Each curve represents a smooth estimate function (loess estimate) of BMI changes over time and the confidence bands are the 95% confidence intervals of the smoothed curve.

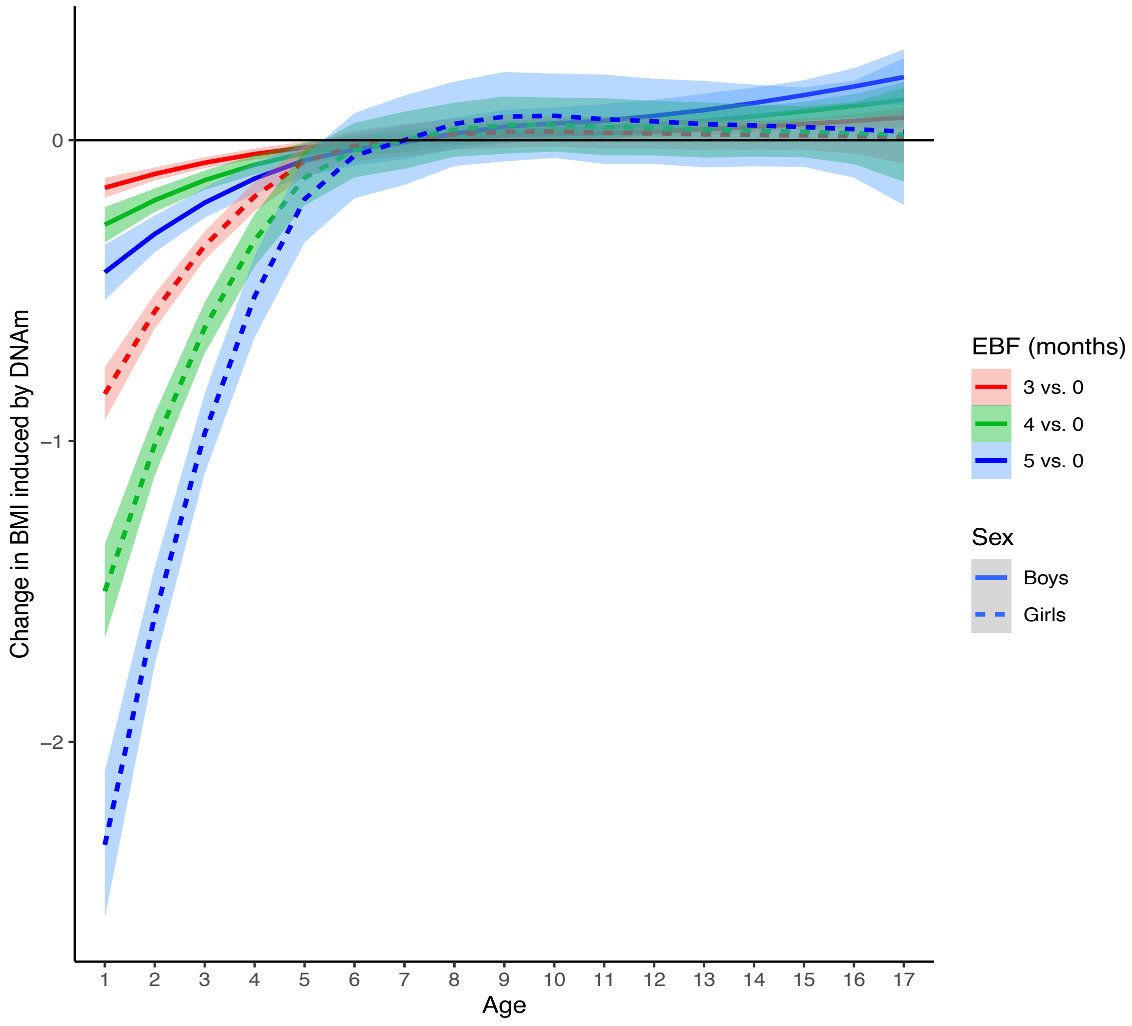

Extended data Fig.6. QQ plots of our test statistic to detect significant CpG sites after adjustment for genomic controls in ALSPAC girls (a) and boys (b). Each plot displays the sample quantiles (y-axis) vs. the theoretical quantiles of a $\chi^2(4)$ distribution (x-axis).

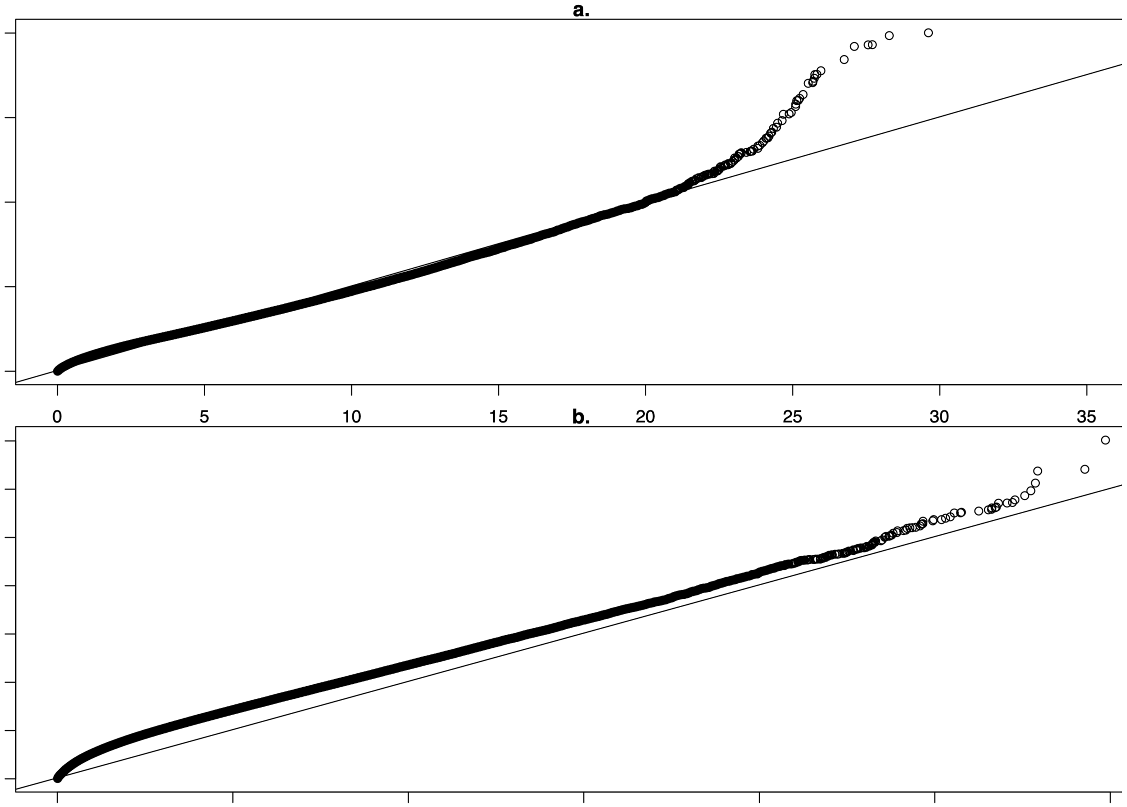

Supplement: Supplementary file 1 — Additional file 1. Supplementary tables and figures. [file 13148_2021_1209_MOESM1_ESM.pdf]
